# Solid state ionics: a Japan perspective

**DOI:** 10.1080/14686996.2017.1328955

**Published:** 2017-07-25

**Authors:** Osamu Yamamoto

**Affiliations:** ^a^ Graduate School of Engineering, Mie University, Tsu, Japan

**Keywords:** Solid state ionics, solid electrolyte, ionic conductor, mixed conductor, diffusion, average structure, solid state battery, sensor, fuel cell, 50 Energy materials, 207 Fuel cells / Batteries / Super capacitors, 206 Energy conversion / transport / storage / recovery

## Abstract

The 70-year history of scientific endeavor of solid state ionics research in Japan is reviewed to show the contribution of Japanese scientists to the basic science of solid state ionics and its applications. The term ‘solid state ionics’ was defined by Takehiko Takahashi of Nagoya University, Japan: it refers to ions in solids, especially solids that exhibit high ionic conductivity at a fairly low temperature below their melting points. During the last few decades of exploration, many ion conducting solids have been discovered in Japan such as the copper-ion conductor Rb_4_Cu_16_I_7_Cl_13_, proton conductor SrCe_1–*x*_Y_*x*_O_3_, oxide-ion conductor La_0.9_Sr_0.9_Ga_0.9_Mg_0.1_O_3_, and lithium-ion conductor Li_10_GeP_2_S_12_. Rb_4_Cu_16_I_7_Cl_13_ has a conductivity of 0.33 S cm^–1^ at 25 °C, which is the highest of all room temperature ion conductive solid electrolytes reported to date, and Li_10_GeP_2_S_12_ has a conductivity of 0.012 S cm^–1^ at 25 °C, which is the highest among lithium-ion conductors reported to date. Research on high-temperature proton conducting ceramics began in Japan. The history, the discovery of novel ionic conductors and the story behind them are summarized along with basic science and technology.

## Graphical abstract

**Figure:**
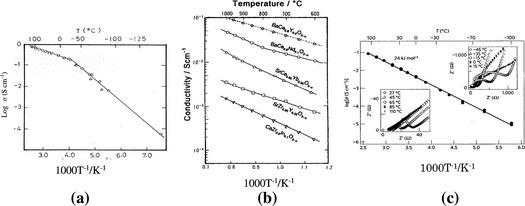

## 1. Introduction

The scientific term ‘ionics’ has been commonly used, from the beginning of electrochemistry, as an indivisible pair of components of the electrochemistry discipline together with ‘electrodics’, and majority of ionics research has been limited to aqueous or liquid electrolytes. Looking back at the history after the pioneering works of Faraday, who found ion transport not only in liquid electrolytes but also in solids of Ag_2_S and PbF_2_, one may find that only little attention has been payed to ‘solid ionics’ materials, in contrast to the rich and variety applications of ‘liquid’ electrochemistry. The disadvantage of low conductivity in electrochemical applications at low temperatures, which has been considered as a detrimental problem, overrides advantages, such as applications at elevated temperatures, possible integration with other solid devices, and so on. From the Nernst grower, the first practical application of solid state ionics materials, to oxygen sensors to monitor oxygen activity in molten metals and exhaust gas from combustion engines, only zirconia-based ceramics materials have been used in limited applications of potentiometric use at high temperatures.

About 130 years after Faraday’s discovery, the term ‘solid state ionics’ was introduced and defined by Takehiko Takahashi of Nagoya University, Japan. He first used the term ‘solid ionics’ in a paper submitted to Denki Kagaku in 1967 [1], the title of which was ‘Study on solid ionics-electrical conductivity of solid electrolyte: electrical conductivity in a Ag_2_S-HgI_2_ system’. The coincidence of the materials used by Faraday’s discovery suggests the presence of limited materials systems that show fast ion conductivity. Later, Professor Takahashi submitted another paper, in 1971, to the *Journal of the Electrochemical Society*. The term, ‘solid ionics’, was slightly modified and this was the very first article that used ‘solid state ionics’ in its title: ‘Solid-state ionics—coulometric titrations and measurements of the ionic conductivity of beta Ag_2_Se and beta Ag_2_Te and use of these compounds in an electrochemical analog memory element’ [2]. Still, the materials are closely related to the one discovered by Faraday. One of the hopes behind the modification of the name to ‘solid state ionics’ includes a wish for ‘solid state ionics’ to become as popular as ‘solid state electronics’, which had been quite rapidly growing, and became a core of the science and engineering field. The term was also used in ‘The Third International Conference on Solid Electrolyte-Solid State Ionics and Galvanic Cells’, held in 1980 in Tokyo, Japan, where the definition of solid state ionics was introduced as the phenomena related to mobile ions in solids; more precisely, ‘solid state ionics’ as a science about solids that can exhibit extraordinarily high ionic conductivity at a fairly low temperature, far below their melting points, and applications as electrochemical devices [3]. After the fourth conference in 1983 in Grenoble, France, the conference name was consolidated as ‘International Conference on Solid State Ionics’, and this series of conference has been held to date. Elsevier launched a new international journal, *Solid State Ionics* in 1980, and the term was thus widely accepted as a branch of solid state science and its applications in international scientific communities.


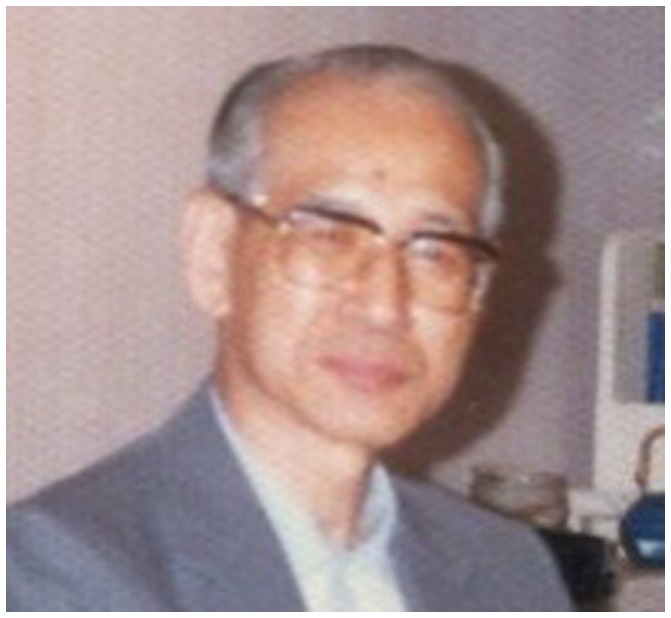


Professor T. Takahashi (1916–1995)

In Japan, the main research activity in the field of solid state ionics (SSI) started in the early 1950s. The early studies focused on the crystal structure analysis and phase transition in highly ion conductive solid electrolytes, such as silver and copper halides and chalcogenides, which exhibit high ionic conductivity at high temperatures. One of the first reports in this field was published in 1952 in *the Journal of the Physical Society of Japan* by Miyake, Hoshino, and Takenaka [4]. In 1966, Yokota proposed a caterpillar mechanism for the correlated diffusion of Ag^+^ ions in silver-ion solid conductors [5]. During the past few decades, many highly ion conductive solid electrolytes were found in Japan; a copper-ion conducting solid Rb_4_Cu_16_I_7_Cl_13_ by Yamamoto and co-workers in 1979 [6], proton conducting oxides of composition SrCe_1-*x*_Y_*x*_O_3_ by Iwahara and co-workers in 1981 [7], oxide-ion conducting solid Sr-doped LaGaO_3_ by Ishihara and co-workers in 1994 [8], and lithium-ion conducting sulfide Li_10_GeP_2_S_12_ by Kanno and co-workers in 2011 [9]. The first domestic solid state ionics symposium was held in 1972 in Nagoya, Japan. Twenty-four papers were presented at this symposium, at which the main research topics were ion conduction mechanisms, the conducting behaviors of Ag^+^, Cu^+^, Na^+^, and O^2-^ ion conductors, and the application of these materials in sensors and batteries. A total of 118 papers were presented at the 38th symposium in 2012, at which the main research topics were electrode materials and electrolytes for lithium batteries and oxide-ion and proton conductors for fuel cells.

One of the dreams for scientists in solid state ionics is still to discover fast, novel, and even exotic ion conductors in the solid state, since most of the solid state ion conductors suffer from low conductivity in comparison with liquid systems owing to slow diffusion kinetics in the solid state. Japanese scientists have been contributing by their achievement in discovering new materials systems as described above and their application as energy and information devices, such as solid oxide fuel cells, NaS battery, Couliode, Memoriode, and so on. This review is aimed to introduce the historical background and recent scientific achievements in the field of solid state ionics in Japan.

## 2. Crystal structure of highly silver-ion conducting solid electrolyte α-AgI

Silver iodide has three solid phases at ambient pressure: body-centered cubic α-AgI, hexagonal β-AgI, and face-centered cubic γ-AgI. The α-phase is stable from 147 °C to its melting point at 555 °C [10], exhibiting an extremely high silver-ion conductivity of about 1.3 to about 2.6 S cm^–1^ that then drops by about 10% upon melting [11]. The conductivity of β-AgI at room temperature is around 10^–6^ S cm^–1^. This low conductivity can be explained by Frenkel defects [12] and silver ion vacancies produced by aliovalent impurities. γ-AgI is metastable at ambient pressure and stable at high pressure; its conductivity at 500 MPa and room temperature has been reported to be 10^–4^ S cm^–1^ [13].

**Figure 1.**
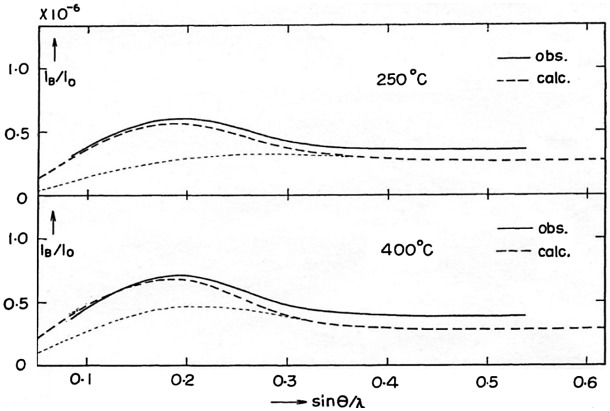
Observed and calculated intensities of the diffuse background in X-ray signals from α-AgI. Reprinted with permission from [15].

**Figure 2.**
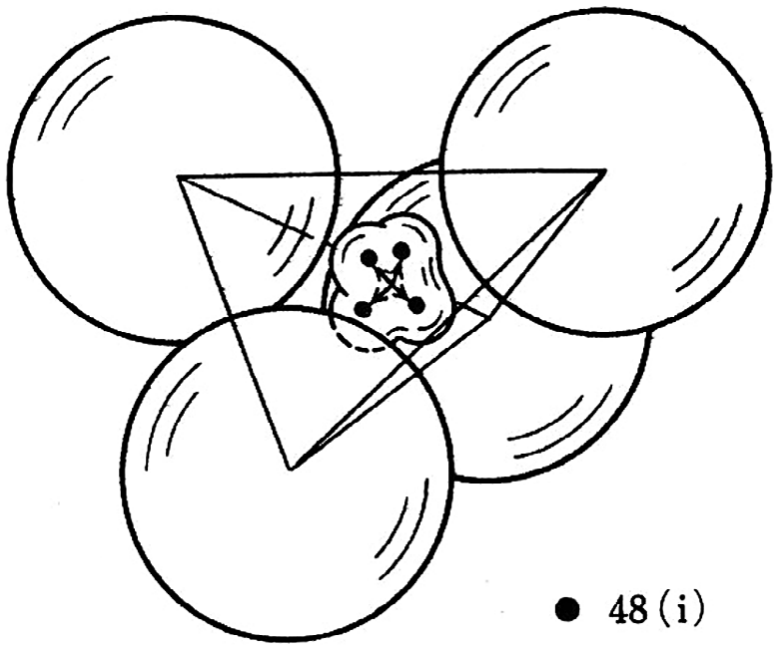
Ag^+^ ion asymmetric anharmonic thermal vibration model of α-AgI. Reprinted from [17] with permission from Elsevier.

The extraordinarily high silver-ion conductivity of α-AgI in the solid phase was explained by its abnormal crystal structure. The crystal structure of the α-AgI phase was first determined in 1934 and then further modified in 1936 by Strock [14]. The structure is famous as a typical example of the ‘average structure’. According to the X-ray diffraction (XRD) analysis by Strock, α-AgI has a cubic unit cell in which two silver atoms are distributed statistically over 42 sites around a body-centered arrangement of iodine atoms. Strock considered that the silver atoms may behave almost liquid-like, in accordance with the remarkably high ionic conductivity of this phase. However, the results were still qualitative. Subsequently, Hoshino [15] measured the XRD intensity of the diffuse background from α-AgI and compared it with that calculated assuming short-range order, and appropriate probability functions for the presence of atoms in different sites. It was confirmed that the intensity of the diffused background (I_B_) measured at 250 °C and 400 °C was in fair agreement with the theoretical intensity in the lower diffraction angle range, as shown Figure 1. The thin dashed curve indicates I_B_+I_o_, where I_B_ is the diffuse background and I_o_ is total intensity flux of the incident beam. The difference between the dashed curve and the dotted curve corresponds to the theoretical value of I_D_. The deviation seemed to result from the rough approximation used in the calculation of I_B_ where the correlation among the displacements of atoms was neglected. Hoshino considered that the liquid-like state of silver atoms suggested by Strock may be a valid picture for the structure of α-AgI in view of its high randomness of silver atoms owing to their statistical distribution over many sites, and the large statistical displacements from the mean positions of atoms. Hoshino et al. [16] also measured only the elastic scattering caused by a disordered distribution of cations in α-AgI type AgI, Ag_3_SI and Ag_2_S using a neutron disuse scattering technique. In 1977, Hoshino et al. [17] reported a powder XRD and neutron diffraction analysis of α-AgI, which showed that two silver ions in the unit cell are distributed over 12(d) sites as a mean with large asymmetric anharmonic thermal vibrations, as shown in Figure 2. This structural model has been supported by the single crystal neutron diffraction study by Cava et al. [18].

## 3. Caterpillar mechanism for diffusion of ions in highly ion conductive solids

In 1966, Yokota [5] proposed a new diffusion mechanism to interpret the remarkable deviation from the Nernst–Einstein relation observed for the diffusion of silver-ions in the high-temperature phases of Ag_2_S, Ag_2_Se, and alloy Ag_2_S_1-*x*_Se_*x*_ reported by Yokota [5] and Okazaki [19]. The diffusion coefficient D of silver-ions in a solid electrolyte is expected to be related to the mobility μ by the Nernst–Einstein relation:(1)
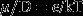



The deviation from the Nernst–Einstein relation was estimated by measuring the drift of silver-ions using an electrochemical ion probe method, and the diffusion coefficient was measured using a tracer diffusion method with the ^110^Ag radioisotope. Okazaki observed a large deviation from equation (1). It is generally considered that a correlation factor *f* should be introduced into the Nernst–Einstein relation:(2)
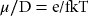



**Figure 3.**
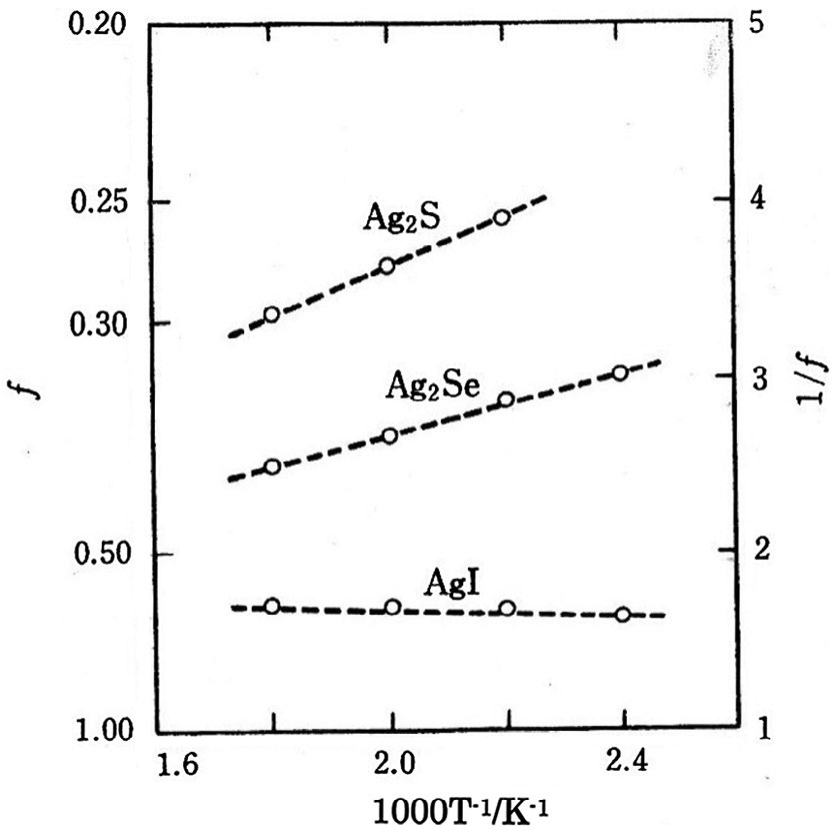
Temperature dependence of the Haven ratio for α-AgI, α-Ag_2_S, and α-Ag_2_Se. Replotted from [19].

*f* is known as the Haven ratio and is dependent on the crystal structure and the conduction mechanism. The meaning of the Haven ratio is best understood in terms of linear response theory as introduced by Kubo in 1957 [20]. For example, the vacancy diffusion is estimated to be 0.78 in the face-centered cubic structure and 0.72 in the body-centered cubic structure [19]. The temperature dependences of the Haven ratio for the highly silver-ion conducting solids α-AgI, α-Ag_2_S, and α-Ag_2_Se are shown in Figure 3. The Haven ratios of α-Ag_2_S and α-Ag_2_Se are remarkably low. Yokota explained the low Haven ratio of these compounds using the caterpillar mechanism.

**Figure 4.**
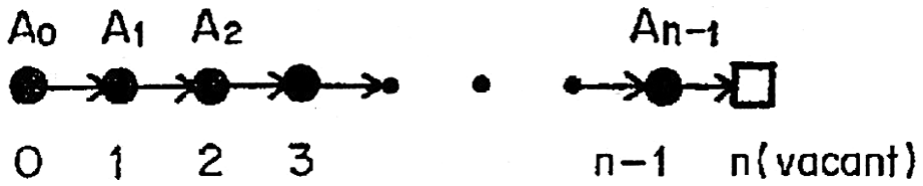
Cooperative jump of ions associated with the spontaneous jump 0→1 of tracer ion A_o_ [5].

The process of diffusion in solids has been interpreted in terms of vacancy or interstitialcy mechanisms, and the deviation from the Nernst–Einstein relation has been attributed to the correlation effect [19–23], i.e. the existence of a correlation between successive jumps of an ion. The correlation effect theory presupposes the solid to be an almost perfect crystal; however, the crystal structures of these highly silver-ion conducting solids are far from perfect. The mobile silver-ions are known to be randomly distributed over the available sites in the crystal lattice [14]. In a highly silver-ion conducting solid such as α-AgI, a low *f*-value must imply that one jump of a tracer ion is accompanied by jumps of adjacent several ions and that the directions of the later jumps are the same as or at least positively correlated with the direction of the initial jump. Yokota proposed a diffusion mechanism that allows such cooperative jumps of several ions. It was supposed that an ion in a site is able to jump not only into a vacant neighboring site but also into an occupied site, which would induce the ion in the latter site to make a jump. The direction of this induced jump will be the same (if crystallographically possible) as or at least positively correlated with the direction of the preceding jump. For simplicity, it was assumed that there are straight lines in the crystal lattices along which sites available to the diffusing ions lie with equal spacing and that the cooperative jumps can take place only along these lines. One of these lines is shown in Figure 4. Suppose that ion A_0_ on site 0 makes a spontaneous jump to site 1, which is neighboring site 0 and is occupied by ion A_1_. Ion A_1_ will be induced to jump to site 2. If site 2 is occupied by ion A_2_, then A_2_ will be induced to jump to site 3. If all the sites from 0 to *n*−1 are occupied by ions, then A_*n*–1_ will be induced to jump to site *n*. Such successive jumps contribute in steps to the electrical conduction, which contributes only one step to the self-diffusion of a specified A_0_ ion. According to this single direction model, the Haven ratio *f* is calculated from a simple equation using an occupancy fraction *P* as(3)
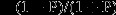



in which it is assumed that there is no difference between the probability per unit time that an ion will make a spontaneous jump to a vacant neighboring site and that to an occupied site. In the case of α-AgI, two Ag^+^ ions are distributed over the 12 (d) sites, but Ag^+^ cannot occupy the nearest neighboring sites, therefore, *P* is 1/3 and *f* is calculated to be 0.5. In the case of α-Ag_2_S, four Ag^+^ ions are distributed over the 12 (d) sites, so *P* is 2/3 and *f* is 0.2. Thus, the Haven ratio was semi-quantitatively explained by this simple theoretical calculation.

## 4. Highly silver-ion and copper-ion conducting solid electrolytes

In the early twentieth century, the electrical conductivities and ionic transport numbers of many types of ionic compounds were measured, and these results were summarized by Tubandt [11]. Almost all of the compounds exhibited low ionic conductivity below their melting temperature, except for AgI, CuBr and CuI. AgI, CuBr and CuI exhibited high silver and copper-ion conductivity at fairly low temperatures below their melting temperatures [11]. The high ionic conductivity of these compounds was explained by their abnormal crystal structure, referred to as the average structure [24]. The possibility of finding other solid electrolytes that possessed high ionic conductivity at ambient temperature was of significant interest. However, no solid electrolyte with the average structure at room temperature was reported until 1961. Reuter and Hardel [25] found that Ag_3_SI had an extremely high silver-ion conductivity of ca. 1 × 10^–2^ S cm^–1^ and no electronic conductivity at room temperature; this value is several orders higher than that of β-AgI. The high ionic conductivity was explained by it having the average structure, which is the existence of considerable ionic probability density along the passageways used by the mobile ions.

**Figure 5.**
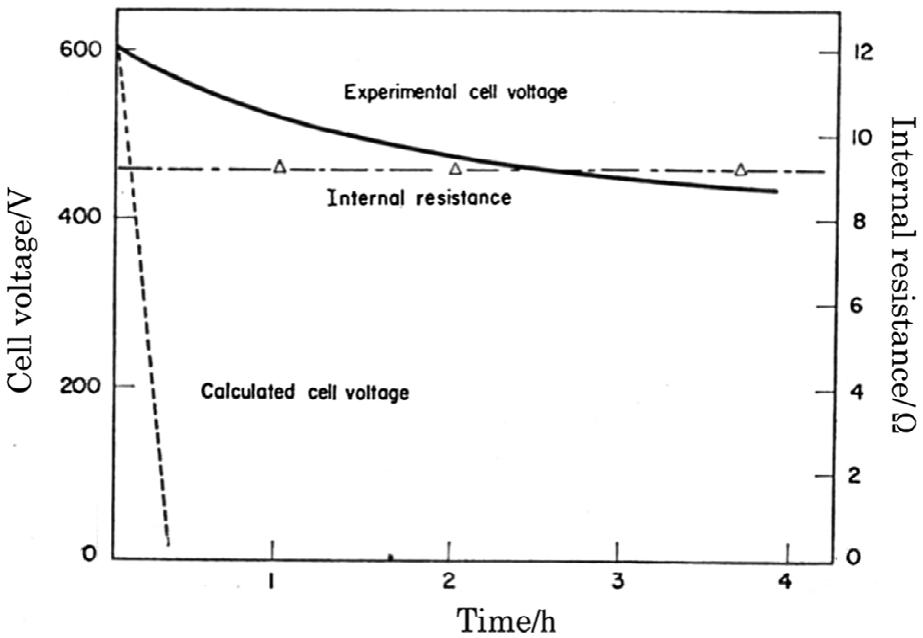
Steady current (1 mA cm^–2^) discharge curve and the change in internal resistance of the Ag-Hg/Ag_3_SI/I_2_-acetylene black cell at 25 °C. Reprinted from [26] with permission from Elsevier.

This high silver-ion conductor was applied as an electrolyte in an all-solid electrolyte cell fabricated by Takahashi and Yamamoto in 1966 [26]. They constructed a cell with a silver amalgam anode, a Ag_3_SI electrolyte, and an acetylene black with I_2_ cathode. The open-circuit voltage of the cell at room temperature was 0.675 V, which was comparable with the theoretical value. The cell showed a low cell voltage drop of 0.1 V after 3 h at 1 mA cm^–2^, as shown in Figure 5. Previously reported all-solid-state cells using AgI could only pass a current of microamperes per cm^2^ because of their high internal cell resistance. The cell with the high silver-ion conducting Ag_3_SI solid electrolyte, the thickness and diameter of which were ca. 0.15 cm and 1.2 cm, respectively, exhibited a low cell resistance of ca. 9 Ω. The internal resistance was constant during discharge, which raised the question as to where the high resistivity products were forming. Takahashi and Yamamoto suggested that the AgI product dissolved in the electrolyte and could not form a resistive phase for the AgI-Ag_2_S system in the solid solution region between the AgI and Ag_2_S. The dashed line in Figure 5 shows the change in cell voltage during the discharge period with the IR drop for the resistance of the reaction product AgI. The cell was able to operate at −17 °C and a current density of several hundred microamperes per square centimeter.

In 1967, Bradley and Greene [27] and Owens and Argue [28] independently reported the isostructural group of compounds MAg_4_I_5_, where M was selected from K, Rb, NH_4_, and to a limited extent Cs. The conductivity of RbAg_4_I_5_, which is 0.26 S cm^–1^ at 20 °C, is comparable to those of aqueous KOH and H_2_SO_4_. Liquid electrolyte solutions are more conductive at room temperature, whereas solid electrolytes have higher conductivity than the liquid electrolyte at and below −40 °C. Therefore, batteries with a solid electrolyte could be especially useful in applications where low-temperature performance is required. Argue and Owens [29] have reported some discharge performance for a Ag/RbAg_4_I_5_/RbI_3_,C cell in the temperature range of −40 to 65 °C. A theoretical capacity of 80% was obtained at current density of up to 40 mA cm^–2^, above which many systems have been shown to develop a highly silver-ion conducting solid electrolyte. However, no highly silver-ion conducting solid electrolyte with conductivity higher than that of RbAg_4_I_5_ has been reported yet.

**Figure 6.**
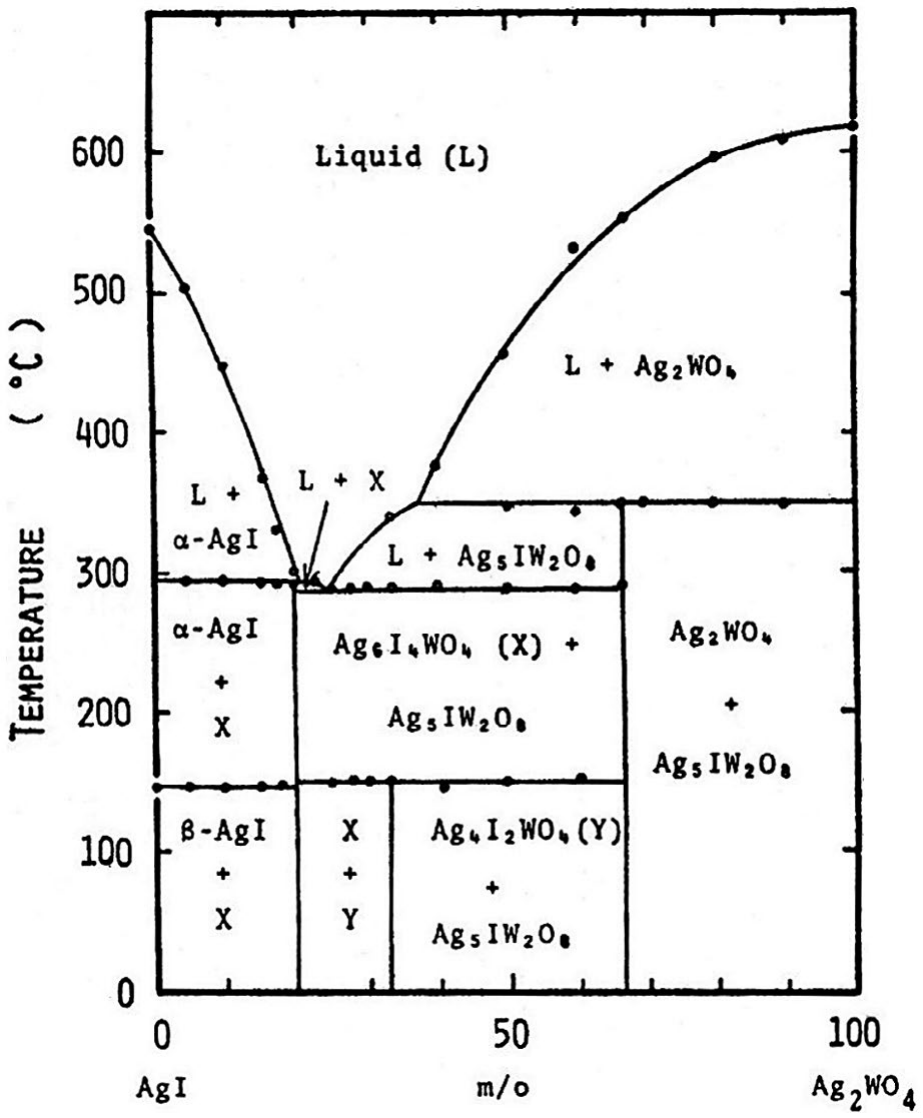
Phase diagram of the AgI-Ag_2_WO_4_ system. Reprinted with permission from *J. Electrochem. Soc.*, **120**, 647 (1973). Copyright 1973, The Electrochemical Society [30].

**Figure 7.**
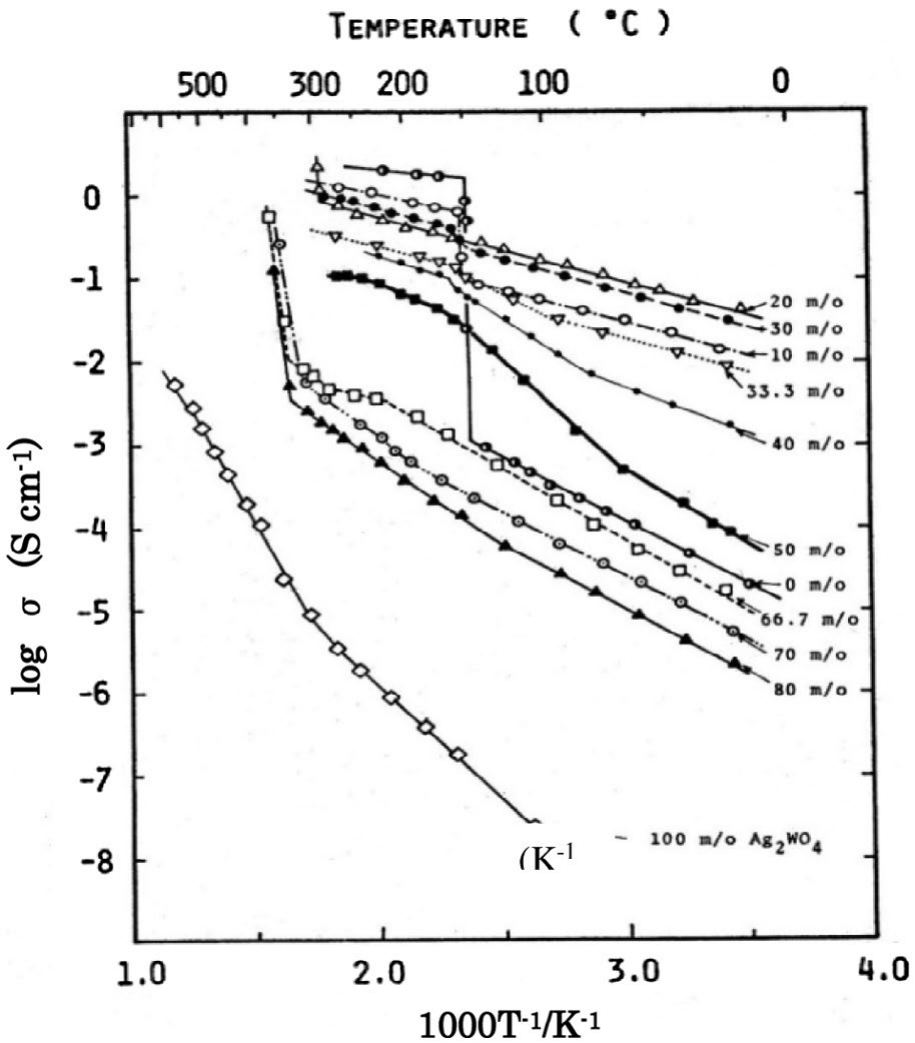
Temperature dependence of electrical conductivity for the AgI-Ag_2_WO_4_ system, where m/o stands for mol%. Reprinted with permission from *J. Electrochem. Soc.*, **120**, 647 (1973). Copyright 1973, The Electrochemical Society [30].

**Figure 8.**
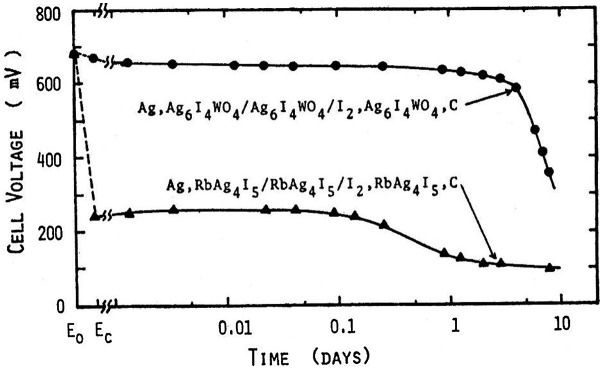
Time dependence of voltage for Ag/Ag_6_I_4_WO_4/_I_2_ and Ag/RbAg_4_I_5_/I_2_ cells at constant load discharge of 7.5 kΩ at 0°C. Reprinted with permission from *J. Electrochem. Soc.*, **120**, 647 (1973). Copyright 1973, The Electrochemical Society [30].

Takahashi et al. [30] reported a new highly silver-ion conducting solid electrolyte stable in ambient atmosphere, because RbAg_4_I_5_ is thermodynamically unstable below 27 °C. They studied the phase diagram of AgI and Ag_2_WO_4_ and found two intermediate compounds, Ag_6_I_4_WO_4_ and Ag_5_I(WO_4_)_2_, as shown in Figure 6. The temperature dependences of the electrical conductivity (*σ*) of the AgI-AgWO_4_ system are shown in Figure 7 as a function of Ag_2_WO_4_ content. The conductivity curves of the samples containing 0 and 10 mol% Ag_2_WO_4_ show an abrupt change at 147 °C, which corresponds to the transition of silver iodide from the β to α-phase. In contrast, that for the 20 mol% Ag_2_WO_4_ sample shows no abrupt change at the same temperature. The ionic conductivity of Ag_6_I_4_WO_4_ was reported to be as high as 0.047 S cm^–1^ at 25 °C, and the compound was stable below room temperature. The discharge curve of the Ag/Ag_6_I_4_WO_4_/I_2_ cell at a constant load of 7.5 kΩ (ca. 0.1 mA cm^–2^) at 0 °C is shown in Figure 8 along with that of the Ag/RbAg_4_I_5_/I_2_ cell. The Ag/Ag_6_I_4_WO_4_/I_2_ cell had an open-circuit voltage of 681 mV and a terminal voltage of 668 mV at the initial stage of discharge, and showed a terminal voltage of 575 mV at 21% anode efficiency. The Ag/RbAg_4_I_5_/I_2_ cell showed a low terminal voltage of 220 mV at the initial stage, because the cell resistance at 0 °C was increased by the decomposition of RbAg_4_I below 27 °C. Takahashi et al. have also reported the discovery of other new highly silver-ion conducting solid electrolytes, Ag_7_I_4_PO_4_ and Ag_10_I_5_P_2_O_7_ [31].

**Figure 9.**
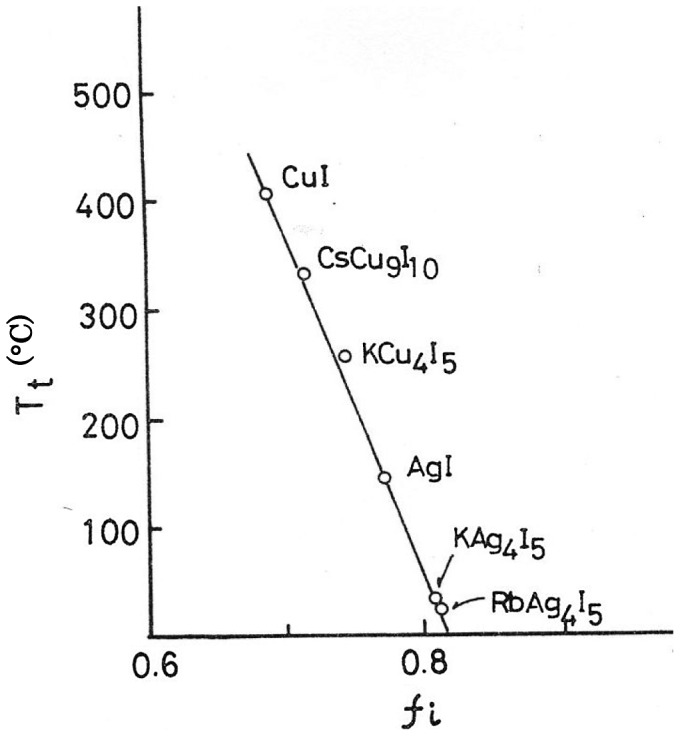
Relationship between ionicity (*f*_i_) and temperature of the phase transition (*T*_t_) to the high ionic conductivity phase. Replotted from [40].

A solid electrolyte, RbAg_4_I_5_, with a high conductivity comparable to that of liquid electrolytes was found in 1967. After that, many systems based on copper (I) halide were examined to find a room temperature highly copper-ion conducting solid electrolyte. The first highly copper-ion conducting solid electrolyte CuBr-C_6_H_12_N_4_CH_3_Br was reported by Takahashi et al*.* in 1973 [32]. The highest copper-ion conductivity was found to be 1.7 × 10^–2^ S cm^–1^ at 20 °C for 0.875CuBr-0.125C_6_H_12_N_4_CH_3_Br. Takahashi et al. [33,34] and Matsui and Wagner studied the phase diagrams of copper (I) halide-alkali halide systems and found many intermediate compounds, but no highly copper-ion conducting solid electrolyte has been observed at room temperature except for 0.75CuCl-0.25RbCl, the conductivity of which is 2.25 × 10^–3^ S cm^–1^ at 25 °C [35]. Phillips proposed that the high ionic conductivity of α-AgI may be related to the crystal bound ionicity (*f*_i_) theory [36], which predicts a 4-fold or a 6-fold coordination for the cations in M^*n*^X^8-*n*^ salts according to whether the ionicity of the crystal bond lies below or above a critical value of 0.785. The ionicity of AgI is 0.770, which is just below the critical value that results in comparable stability of the Ag ions in 4-and 6- coordination and provides large validity for low symmetry scaffold structures. The formation of the highly ion conductive double salt MAg_4_I_5_ (M = K or Rb) can be explained theoretically: the addition of a high ionic salt such as KI or RbI to AgI increases the ionicity of AgI slightly, to near the critical value. Further, at high temperature, CuI transforms into a phase that has a high copper-ion conductivity at 408 °C. The ionicity of CuI is 0.692, and the double salt KCu_4_I_5_ has a high copper-ion conductivity at 257 °C [37]; the ionicity of CuI increases upon the addition of KI and the highly ion conductive compound appears at a temperature below 408 °C. The mean ionicity of the compounds that exist in the copper halide-alkaline halide and silver halide-alkaline halide systems are summarized in Table 1. The mean ionicity of the double salts is taken as the algebraic means of the ionicity of the copper (I) or silver halides and alkaline halide as a zero approximation. The ionicities of these compounds are taken from the values reported by Phillips [38]. The high and low conductivity labels in this table indicate that the conductivity of the compound is higher or lower than 10^–2^ S cm^–1^ at temperatures below its melting point, respectively. The relationship between ionicity and conductivity suggests that the ionicities of all the high conductivity double salts should be less than 0.809. However, not all of the double salts with ionicity lower than 0.809 exhibit high conductivity: CsCu_2_Br_3_ (*f*_i_ = 0.809) and RbCu_3_I_4_ (*f*_i_ = 0.779) have low conductivity. Thus, a critical ionicity value is a necessary requirement to obtain a high conductivity double salt of copper and silver halide systems. Furthermore, Table 1 shows that the high conductivity double salts with ionicity near 0.80 exist at around room temperature while those with lower ionicity such as 0.75 or 0.72 exist only at higher temperatures. Figure 9 shows the relationship between the ionicity and the temperature of the phase transition to the high conductivity phase. The phase transition temperature decreases linearly with increasing ionicity, and the critical ionicity at room temperature is 0.808. These results may provide some information to assist in the search for highly ion conductive double salts.

**Table 1.** Mean ionicity (*f*_i_) and conductivity of compounds in the silver halide-alkali halide and copper (I) halide-alkali halide systems [40].

Compound	Stability range ( °C)	Conductivity	*f*_i_	References
Rb_2_AgBr_3_	<310	low	0.921	28
CsAgBr_2_	<265	low	0.904	28
Cs_2_AgBr_3_	<275	low	0.921	28
KAg_4_I_5_	38–253	high	0.806	40
K_2_AgI_3_	<130	low	0.890	40
RbAg_4_I_5_	27–228	high	0.807	28, 29
Rb_2_AgI_3_	<298	low	0.892	28
CsAg_2_I_3_	<210	low	0.831	28
Cs_2_AgI_3_	<253	low	0.892	28
K_2_CuCl_3_	<148	low	0.884	41
Rb_3_Cu_7_Cl_10_	<165	high	0.809	42
Rb_2_Cu_3_Cl_5_	<187	low	0.830	42
Rb_2_CuCl_3_	<205	low	0.902	42
CsCu_2_Cl_3_	<277	low	0.816	42
Cs_3_Cu_2_Cl_5_	<310	low	0.871	42
K_2_CuBr_3_	<228	low	0.880	43
Rb_2_CuBr_3_	<251	low	0.883	42
CsCu_9_Br_10_	290–306	high	0.757	42
CsCu_2_Br_3_	<355	low	0.809	42
KCu_4_I_5_	257–332	high	0.745	38, 41
RbCu_2_I_3_	<285	low	0.779	28
Rb_2_CuI_3_	<298	low	0.866	31
CsCu_9_I_10_	333–348	high	0.718	42
CsCu_2_I_3_	<348	low	0.779	42
Cs_3_Cu_2_I_5_	<352	low	0.849	39

**Figure 10.**
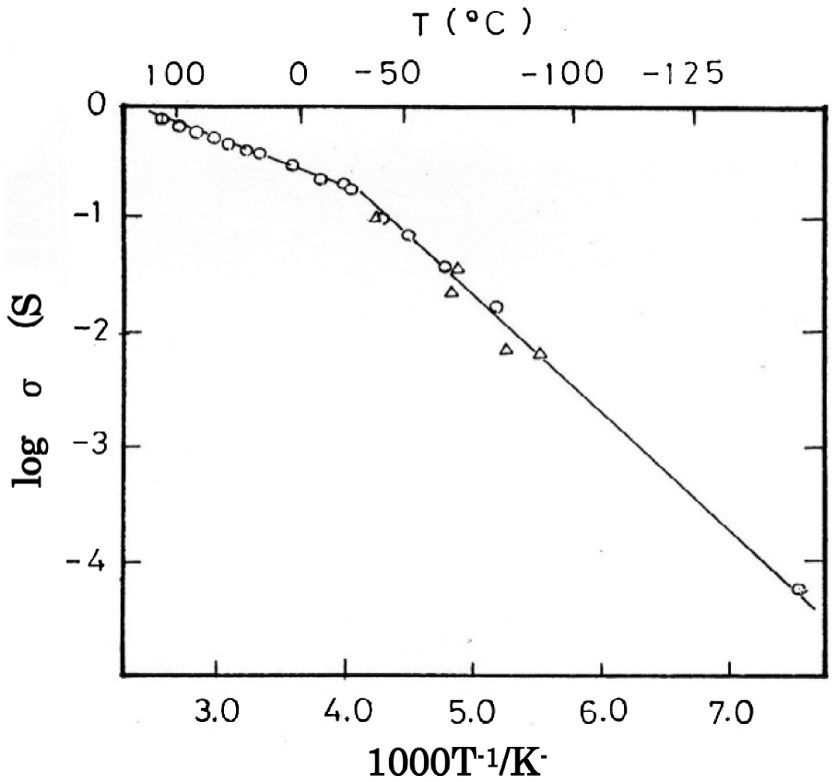
Temperature dependence of the conductivity (*σ*) of Rb_4_Cu_16_I_7_Cl_13_. ○: Cooling; Δ: heating. Reprinted with permission from *J. Electrochem. Soc.*, **126**, 1654 (1979). Copyright 1979, The Electrochemical Society [6].

Kanno et al. [41] reported that Rb_3_Cu_7_Cl_10_ has a relatively high copper-ion conductivity of 3.32 × 10^–3^ S cm^–1^ at room temperature and an iconicity of 0.809, which is similar to that of RbAg_4_I_5_; however, its conductivity is two orders of magnitude lower than that of RbAg_4_I_5_. No new high-conductivity compounds have been found in the other copper (I) halide and alkaline halide binary systems. Meanwhile, Takahashi et al. searched ternary systems of copper (I) halide and alkaline halide according to the above ionicity considerations. In 1979, Takahashi et al. [6] reported a highly copper-ion conducting solid electrolyte at room temperature in the CuCl-CuI-RbCl system. The highest electrical conductivity in this system was found for 0.45CuCl-0.35CuI-0.20RbCl, 3.4 × 10^–1^ S cm^–1^ at 25 °C. XRD results indicated that a sample of Rb_4_Cu_16_I_7_Cl_13_ in composition was a single phase and all the diffraction lines were indexed to a cubic symmetry with a lattice parameter of 1.002 nm. The X-ray and observed densities suggested the chemical formula to be Rb_4_Cu_16_I_7_Cl_13_ per unit cell. The number of constituent atoms in the unit cell coincides with that of RbAg_4_I_5_, which has four entities in a unit cell with *a* = 1.124 nm. These results suggested that the structures of RbAg_4_I_5_ and Rb_4_Cu_16_I_7_Cl_13_ are probably closely related. Figure 10 shows the temperature dependence of the electrical conductivity of Rb_4_Cu_16_I_7_Cl_13_ from −140 to 130 °C. The conductivities in the cooling and heating cycles are in good agreement with each other. This conductivity versus temperature plot shows a transition at approximately −25 °C. A similar conductivity knee was observed for RbAg_4_I_5_ at −64 °C, which undergoes a high order phase transition [28]. The activation energy for conduction was found to be 7.0 kJ mol^–1^ in the high-temperature range and 19 kJ mol^–1^ in the low-temperature range. The powder XRD patterns of Rb_4_Cu_16_I_7_Cl_13_ at room temperature and −100 °C were compared and found to be in good agreement; except for a change in the relative intensity of each peak, no extra peaks or peak splitting were observed. This result suggests that the transition at −25 °C is a high order phase transition, i.e., Rb_4_Cu_16_I_7_Cl_13_ has a high disorder structure above −25 °C and a low disorder structure below −25 °C. Atake et al. [43] measured the heat capacity of Rb_4_Cu_16_I_7_Cl_13_ between 10 and 300 K using a high precision adiabatic calorimeter. An abnormal increase was observed in the heat capacity curve above 90 K, and above 130 K the heat capacity exceeded the classical limit value of 120 R = 998 J K^–1^ mol^–1^. The excess heat capacity showed a broad anomaly with a maximum at about 150 K, which indicates a non-cooperative mechanism for the redistribution of Cu ions among the crystal sites. The abnormal excess heat capacity corresponds to the remarkable variation in the occupancy of the 8-fold site, as shown by the neutron diffraction results reported by Kanno et al*.* [44]. The physical properties of Rb_4_Cu_16_I_7_Cl_13_ are compared with those of RbAg_4_I_5_ [45–48] in Table 2. The copper ion conductivity of 3.4 × 10^–1^ S cm^–1^ at 25 °C is as high as that of an aqueous electrolyte solution, and is still the highest of all room temperature ionic conducting solid electrolytes.

**Table 2.** Physical properties of Rb_4_Cu_16_I_7_Cl_13_ and RbAg_4_I_5_.

	Rb_4_Cu_16_I_7_Cl_13_	RbAg_4_I_5_	References
Crystal structure	Cubic *a* = 1.002 nm	Cubic *a* = 1.124 nm	6, 46
X-ray density (g cm^–3^)	4.47	5.38	6, 46
Electrical conductivity at 25 °C (S cm^–1^)	0.34	0.28	6, 29
Activation energy for conduction (kJ mol^–1^)	7.0	7.1	6, 47
Electronic conductivity (S cm^–1^)	10^–12^ at 60 ℃	10^–11^ at 25 ℃	6, 48
Copper ion or silver ion transport number	1.0	1.00	6, 49
Decomposition potential (V)	0.69	0.67	6, 49
Melting point (℃)	234 (incongruent)	228 (incongruent)	6, 28

## 5. Ion and electron mixed conductors

**Figure 11.**
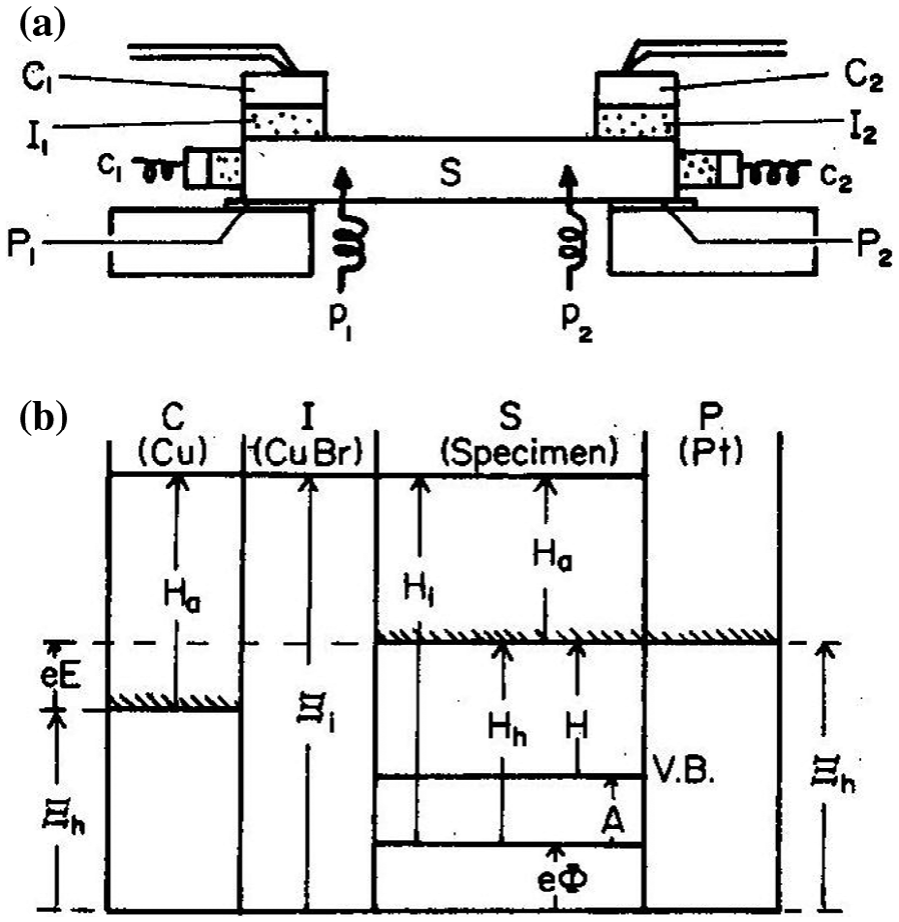
(a) Schematic of the arrangement used for mixed conductivity measurements; I: ionic conductor, S: sample, P: platinum, C: copper or silver; and (b) schematic energy diagram of the Pt/sample/Cu or Ag galvanic cell. Reproduced with permission from [53].

The ionic conductivity of the α-Ag_2_S group (the high-temperature phase of Ag_2_S, Ag_2_Se, and Ag_2_Te), which exhibits mixed conduction of electrons and silver-ions, has been extensively studied by Miyatani and Yokota using an ionic probe [49–51]. The basic concept was proposed by J.B. Wagner and C. Wagner [52]. Figure 11(a) shows a typical setup used to measure the electronic and ionic conductivity separately, where P is platinum, S is the sample, I is the ionic conductor and C is copper (for copper conductors, or silver for silver conductors). By sending a current across C and P, the Cu content in the sample can be varied and the change determined according to Faraday’s law. The electronic conductivity of the sample can be determined by measuring the potential drop between two metal probes, P_1_ and P_2_, by sending a current through the metallic electrodes, while the ionic conductivity can be determined by measuring the potential drop between two ionic probes of I_1_ and I_2_.

**Figure 12.**
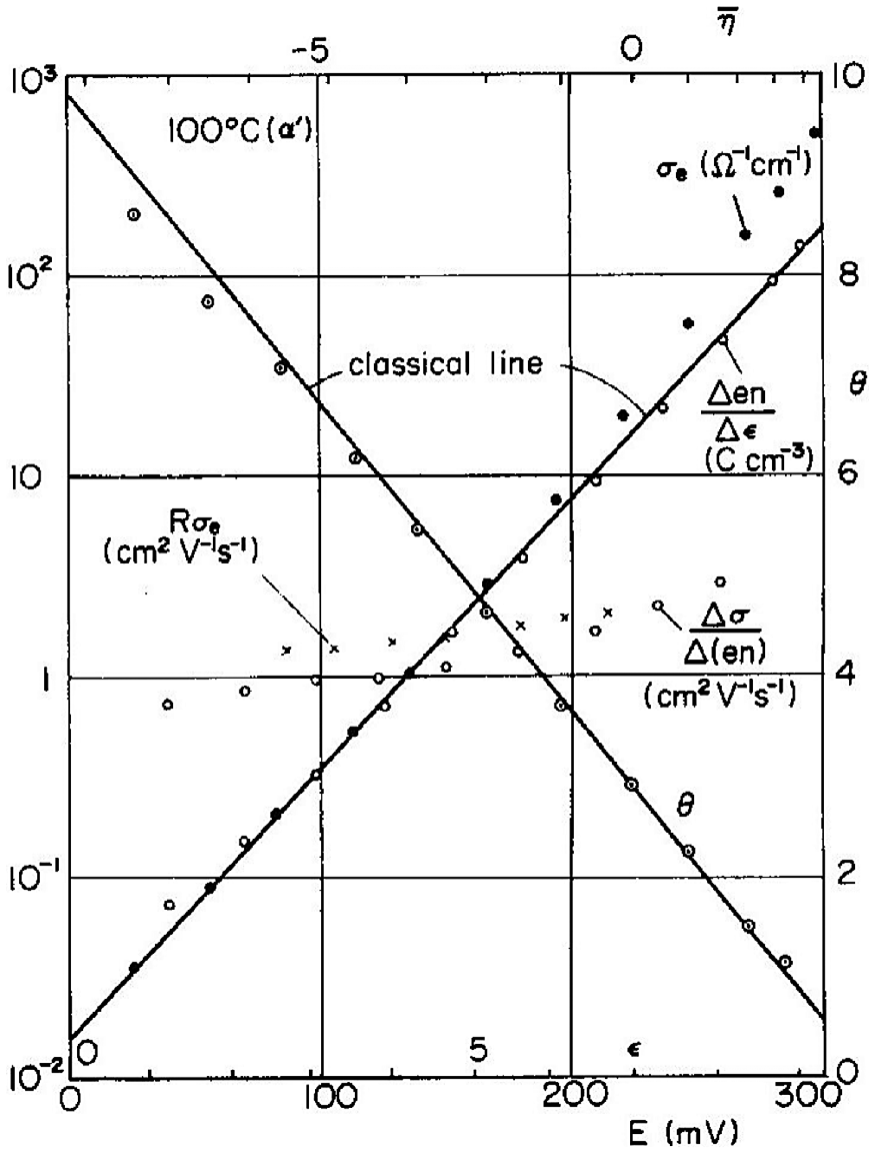
*σ*_e_, Δ(*en*)/Δ*ε*, Δ_e_/Δ(*en*), R*σ*_e_, and *θ* versus *E* curves for α-Cu_2-*x*_S at 100 °C. Reproduced with permission from [53].

Figure 11(b) shows the schematic energy diagram of the galvanic cell Pt/sample/Cu or Ag, where H is the chemical potential, Ξ is the electrochemical potential, *φ* is the electrostatic potential, and suffixes i and h refer to Cu^+^ (or Ag^+^) ion and hole, respectively. Ishikawa and Miyatani measured the electronic and ionic conductivity, Hall coefficient, and thermoelectric power of the electron and ion mixed conductors as a function of the electromotive force (E) for Ag/AgI/silver chalcogenide/Pt and Cu/copper ion conductor/copper chalcogenide/Pt cells, where E may be considered as the relative potential of the Fermi level in the chalcogenides. Figure 12 shows *σ*_e_, Δ(*en*)/Δ*ε*, Δ_e_/Δ(*en*), R*σ*_e_, and *θ* versus E curves for α-Cu_2-*x*_S at 100 °C, where *σ*_e_ is the electronic conductivity, *n* is the number of electrons, *ε* is the dielectric constant, and *θ* is the thermoelectric power. Ishikawa and Miyatani [53] also proposed a model where *E* does not represent the relative value of the Fermi energy and instead they assumed that *E* represents the height of the Fermi level, although the measurement of the surface potential fails to give convincing evidence for this assumption. Therefore, we consider the position of the bottom of the conduction band to be dependent on the increasing excess Ag content in α-Ag_2_S [50] as well as the excess Cu content in Cu_2-*x*_Se, Cu_2-*x*_S, and C_2-*x*_(S, Se) [53]. The extent of the Ag or Cu excess can be interpreted as arising from the screening effect of carriers (holes) on copper or silver ion vacancies.

A theoretical description of mixed electron and ion conductors was made by Wagner [54]. However, his theory is limited to the equilibrium and steady states. The theory of the transition phenomena characteristics of mixed conductors was presented by Yokota and Miyatani [55–57]. The one-dimensional transport of charge carriers in mixed conductors can be described using the following transport equations [58]:


(4)
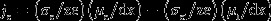




(5)
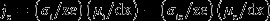



where *j*_e_ and *j*_i_ are the electronic and ionic current, respectively, and *σ*_ei_ and *σ*_ie_ are the respective cross-conductivities. According to the Onsager reciprocal relation [59],(6)
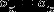



**Figure 13.**
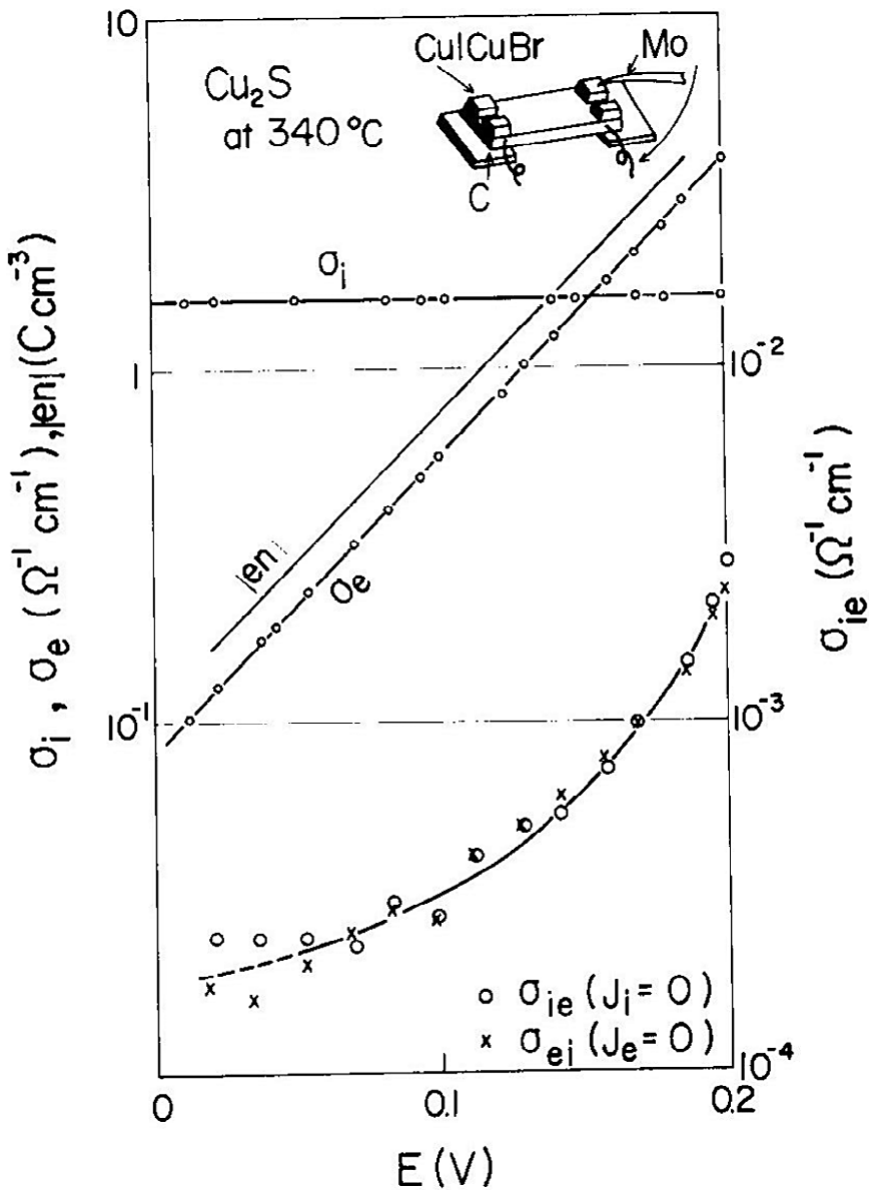
Observed values of cross-conductivity *σ*_ei_ and *σ*_ie_ of Cu_2-*x*_S plotted against E for the Cu_2-*x*_S/CuBr/Cu cell. Reprinted from [56] with permission from Elsevier.

Yokota and Miyatani [56] measured *σ*_ei_ and *σ*_ie_ independently using the ionic and electronic electrode showed in Figure 11. The experimental results for Cu_2-*x*_S at 340 °C are shown in Figure 13, where the abscissa is the electromotive force (emf) *E* for the Cu/Cu_2-*x*_S/CuBr/Cu cell.

**Figure 14.**
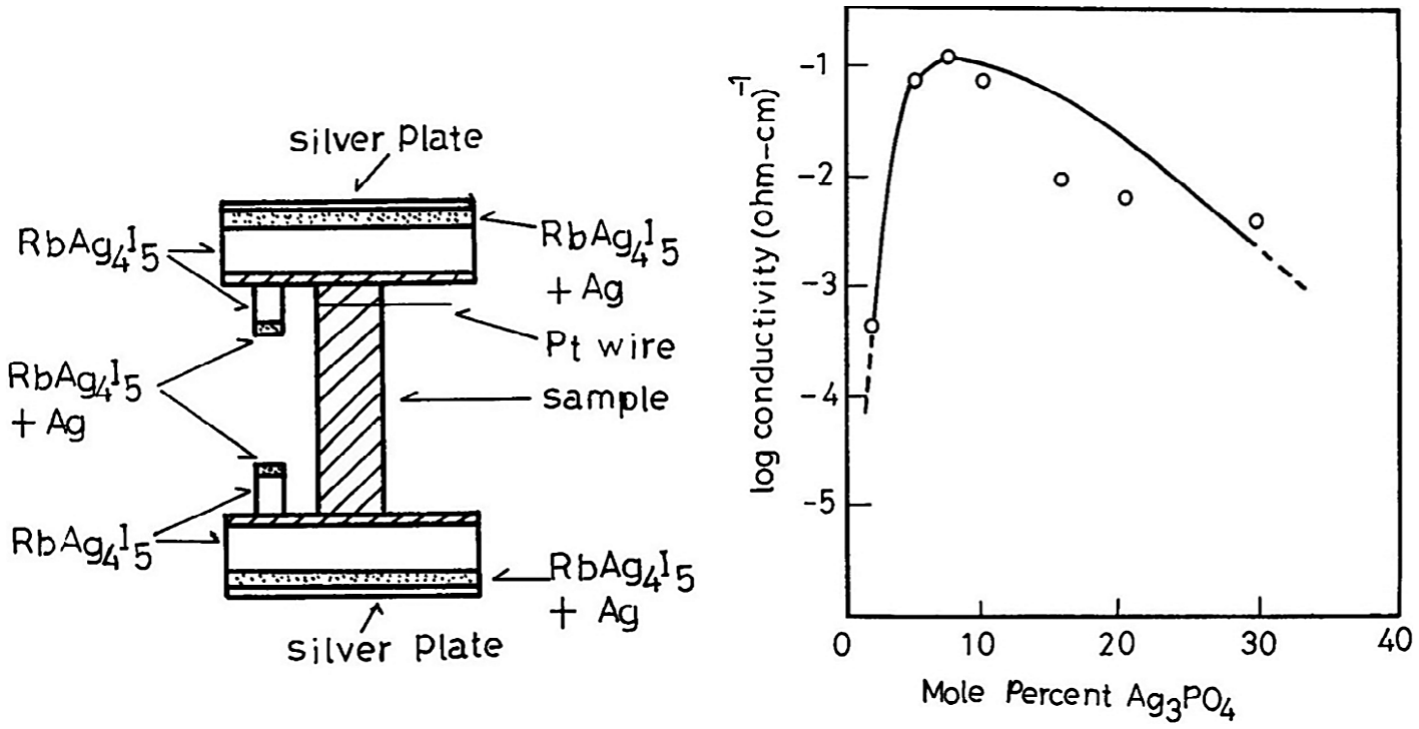
Ionic conductivity measurement setup and ionic conductivity at 30 °C as a function of Ag_3_PO_4_ content for Ag_2_Se-Ag_3_PO_4_. Reprinted with permission from *J. Electrochem. Soc.*, **119**, 1735 (1972). Copyright 1972, The Electrochemical Society [60].

Mixed conductors with high ionic conductivity were found among the high-temperature phases of silver and copper chalcogenides. Takahashi and Yamamoto reported Ag_2_Se–Ag_3_PO_4_ solid solution to be a mixed conductor with high silver-ion conductivity at room temperature [60]. Solid solutions in the Ag_2_Se-Ag_3_PO_4_ system containing 5–10 mol% Ag_3_PO_4_ have the high-temperature cubic α-Ag_2_S structure and high silver-ion conductivity at room temperature. The silver-ion conductivity was measured using the electron blocking cell shown in Figure 14. The ionic conductivity of the solid solution was high so a cylindrical sample 2–3 mm in diameter and 12–13 mm long was used, and Ag/RbAg_4_I_5_ probes were used for potential difference measurements. The effects of the electrode polarization were negligible using this arrangement.

The silver-ion conductivity measured at 30 °C is shown as a function of Ag_3_PO_4_ content in Figure 14. The ionic conductivity increased with the content of Ag_3_PO_4_ in the Ag_2_Se up to 10 mol% and then decreased. Measurement of the lattice constants of the solid solution suggested that the upper limit of Ag_3_PO_4_ content in Ag_2_Se-Ag_3_PO_3_ was approximately 10 mol%. Samples containing greater than 10 mol% Ag_3_PO_4_ were a mixture of the solid solution and Ag_3_PO_4_. The silver-ion conductivity of 0.9Ag_2_Se-0.1Ag_3_PO_4_ was as high as 0.1 S cm^–1^ at 30 °C, which is comparable to that of RbAg_4_I_5_. The activation energy for silver-ion conduction in this solid solution was estimated to be 4.0 kJ mol^–1^ from the temperature dependence of the silver ion conductivity. A non-stoichiometry of Ag in this solid solution was observed in the range of 0 to 0.002 in Ag_2.075-*x*_Se_0.925_(PO_4_)_0.075_ and the activity of silver was changed with the non-stoichiometry. The electrode potential of (Ag_2_Se)_0.925_(Ag_3_PO_4_)_0.075_ was changed from 0 to 150 mV by removing Ag from the solid solution. Electrochemical potential memory devices were developed by Ikeda and Tada [61] using this mixed conductor and the stable silver-ion conductor Ag_6_I_4_WO_4_.

**Figure 15.**
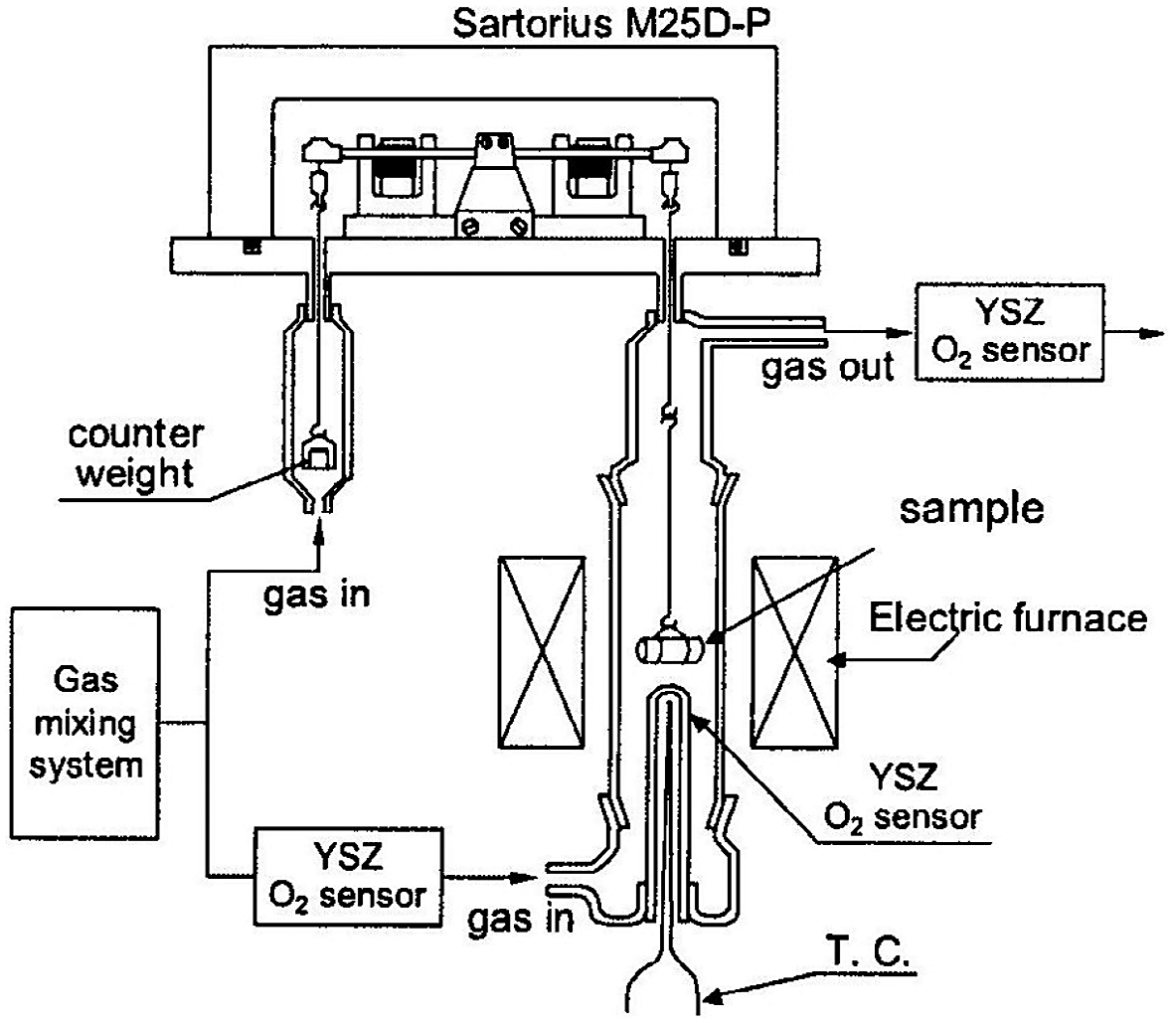
Schematic of the thermogravimetric measurement system. Reprinted from [70] with permission from Elsevier.

**Figure 16.**
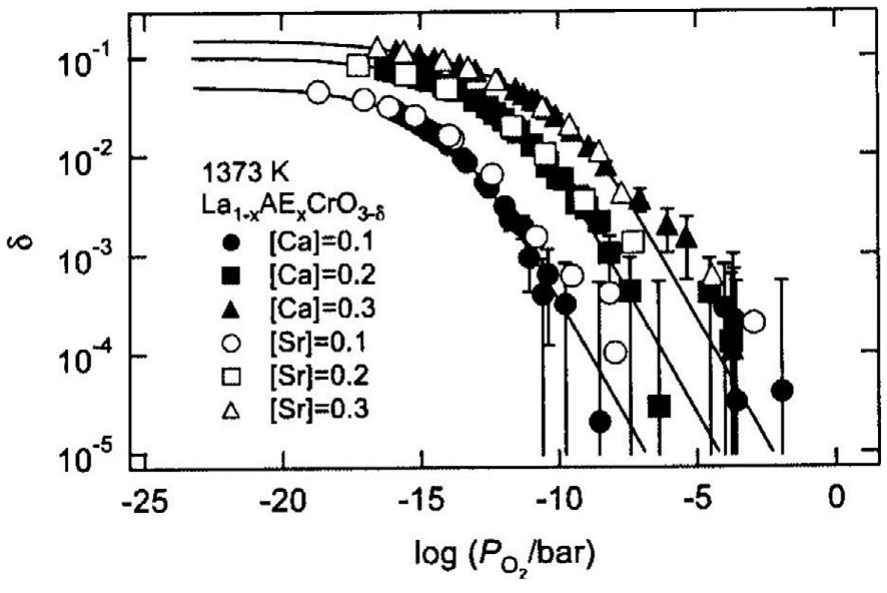
Oxygen non-stoichiometry of La_1-*x*_Ca_*x*_CrO_3-δ_ and La_1-*x*_Sr_*x*_CrO_3-δ_. at 1377 K as a function of log P(O_2_). The fitting results for La_1-*x*_Ca_*x*_CrO_3-δ_ are shown as solid lines. Reprinted from [70] with permission from Elsevier.


Doped perovskite-type oxides of composition La_1-*x*_AE_*x*_MO_3-*δ*_ (AE = Ca, Sr, M = Cr, Mn, Fe, Co) have large oxygen deficiency (oxygen non-stoichiometry), high oxide-ion diffusion and high electronic conductivity [62–64]. Mizusaki and his co-workers at Tohoku University [63,64] extensively studied the oxygen non-stoichiometry and thermo-chemical stability of these oxides as a function of temperature and oxygen partial pressure using thermos-gravimetry and coulometric titration. These perovskite-type oxides have been investigated for applications such as solid oxide fuel cells (SOFCs), gas separators, and oxygen sensors [65–67]. Ishigaki et al. [63] reported that the oxygen non-stoichiometry of La_1-*x*_Sr_*x*_CrO_3-*δ*_ can be described not by an ideal solution model, but by a regular solution model that takes the effect of defect interaction into account. Yasuda and Hikita [68] and Boroomand et al. [69] reported that defect formation in La_1-*x*_Ca_*x*_CrO_3-*δ*_ can be explained with the ideal solution model. Onuma et al. [70] precisely determined *δ* for La_1-*x*_Ca_*x*_CrO_3-*δ*_ as a function of composition, temperature, and P(O_2_) using a thermogravimetric technique to elucidate a suitable defect chemical model for this solid solution. Figure 15 shows a schematic of their thermogravimetric measurement system. In the system, a sample rod was suspended from the electrical microbalance with alumina sticks, quartz sticks, and Pt-Rh wire. The temperature around the sample was controlled within 1 K. The weight change caused by oxygen non-stoichiometry of the La_1-*x*_Ca_*x*_CrO_3-*δ*_ was measured under controlled oxygen partial pressure using O_2_-Ar, CO-CO_2_, and H_2_-H_2_O-Ar mixtures. Three yttria-stabilized zirconia (YSZ) oxygen sensors were installed to monitor P(O_2_). Figure 16 shows the oxygen non-stoichiometry of La_1-*x*_Ca_*x*_CrO_3-*δ*_ and La_1-*x*_Sr_*x*_CrO_3-*δ*_ as a function of log P(O_2_) at 1373 K [70]. There is almost no difference between La_1-*x*_Ca_*x*_CrO_3-*δ*_ and La_1-*x*_Sr_*x*_CrO_3-*δ*_, which means that *δ* is not dependent on the A-site dopant species, but on the dopant concentration. The exchange of oxygen between La_1-*x*_Ca_*x*_CrO_3-*δ*_ and gas phase can be presented using the Kröger–Vink notation:

(7)
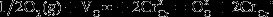



The equilibrium constant for reaction (7), *K*, is


(8)
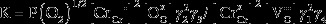



where *γ*_1_, *γ*_2_, *γ*_3_ and *γ*_4_ are the activity coefficients of Cr_Cr_^*x*^, Cr_Cr_^•^, O_O_^*x*^, and V_O_^••^, respectively. The solid curves in Figure 16 represent the fitting results, which agree well with the experimental data. The *K* values of a non-ideal solution tend to be smaller than those reported in the literature because Yasuda and Hikita [68] and Boroomand et al. [69] assumed the defect formation occurred as in an ideal solution.

**Figure 17.**
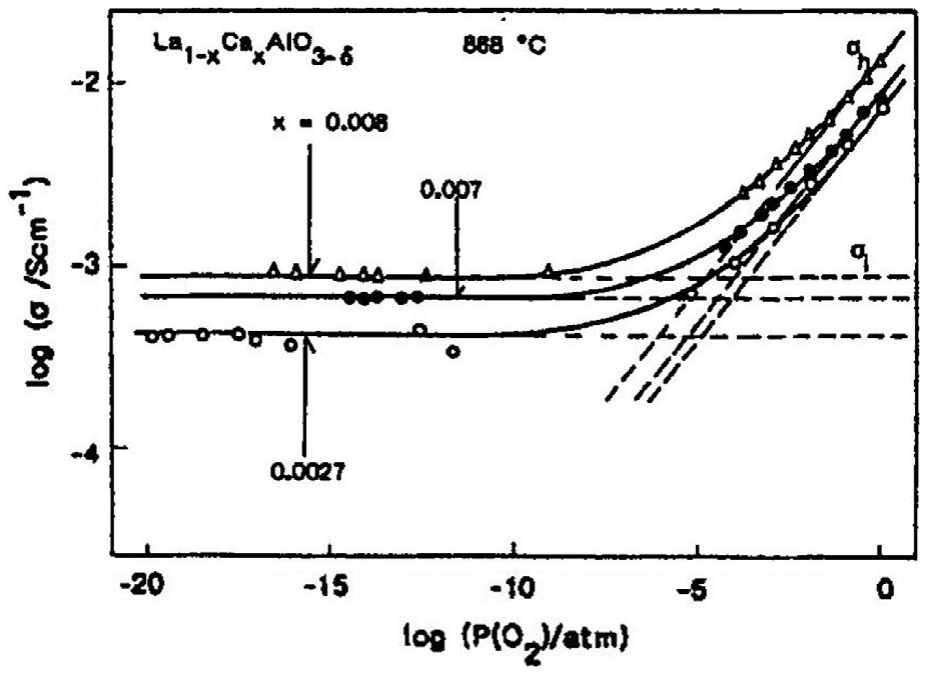
P(O_2_) dependence of conductivity at 868 °C for single crystals of La_1-*x*_Ca_*x*_AlO_3-δ_ with different values of *x*. Reprinted with permission from *J. Electrochem. Soc.*, **140**, 467 (1993). Copyright 1993, The Electrochemical Society [71].

Mizusaki et al. also determined the ionic and electronic conductivity of oxide-ion and electron mixed conductors of oxygen partial pressure using high-temperature gravimetry and DC conductivity measurements as a function [71]. Figure 17 shows a typical example of the oxygen partial pressure dependence of the conductivity of a single crystal of composition La_1-*x*_Ca_*x*_AlO_3-*δ*_, which was reported as a typical perovskite-type oxide-ion conductor by Takahashi and Iwahara [72,73]. The conductivity is essentially constant, irrespective of P(O_2_) at lower P(O_2_). With increasing P(O_2_), the conductivity gradually increases asymptotically approaching the P(O_2_)^1/4^ relationship, which suggests that p-type conduction becomes predominant with increasing P(O_2_). The equilibrium between the gas phase and La_0.995_Ca_0.005_AlO_3-δ_ can be expressed as(9)
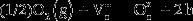



The total conductivity *σ* can be expressed as


(10)
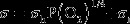



where *σ*_h_ and *σ*_i_ are the hole conductivity and ion conductivity, respectively, and *σ*_i_ can be expressed as


(11)
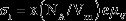



where *N*_A_ is the Avogadro number, *V*_m_ is the molar volume, and *μ*_V_ is the mobility of an oxygen vacancy. *σ*_i_ is expected to be proportional to *x* and dependent on P(O_2_). The best fitting curves are shown in Figure 17.

**Figure 18.**
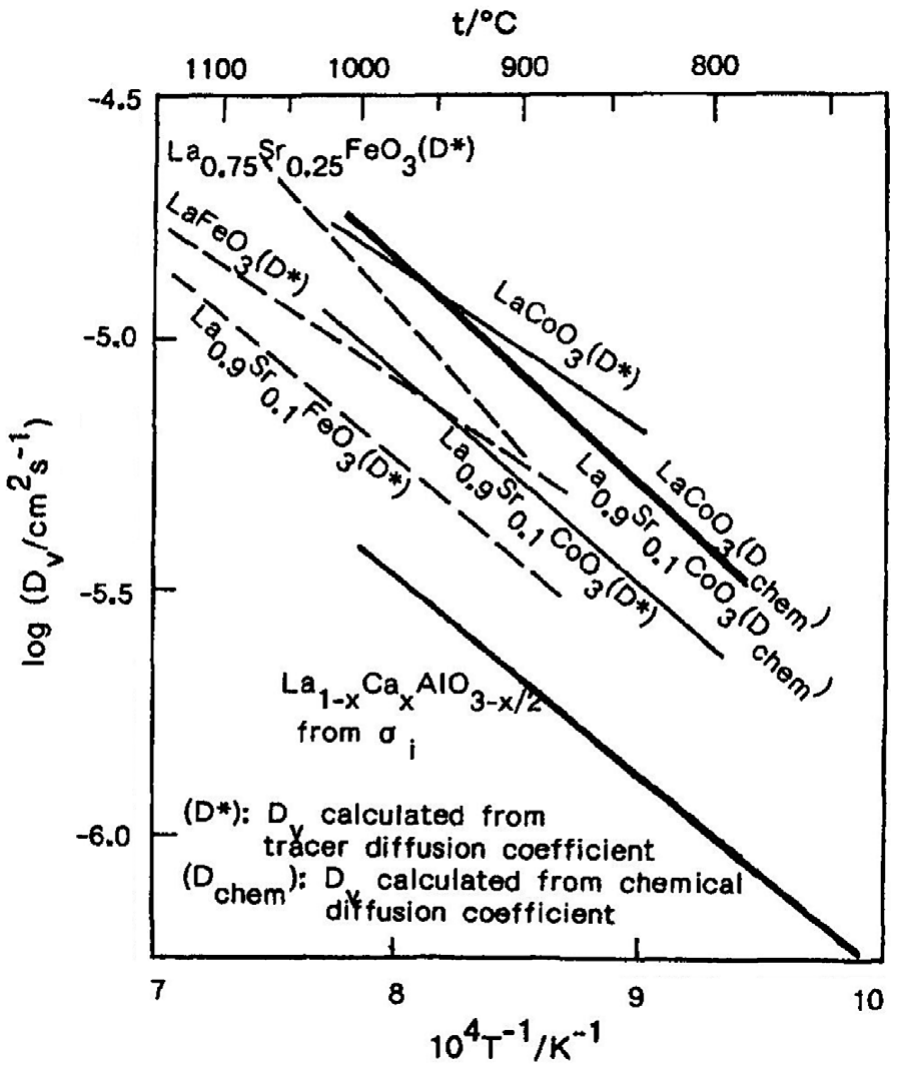
Log D_V_ versus temperature curves for the perovskite-type oxides. Reprinted with permission from *J. Electrochem. Soc.*, **140**, 467 (1993). Copyright 1993, The Electrochemical Society [71].

Fueki et al. measured the oxygen vacancy diffusion coefficient of some perovskite-type oxides, using the chemical relaxation method for La_1-*x*_Sr_*x*_CoO_3-*δ*_ [74] and the isotopic exchange reaction for La_1-*x*_Sr_*x*_CoO_3-*δ*_ [63,75,76] and La_1-*x*_Sr_*x*_FeO_3-*δ*_ [76]. Figure 18 shows the temperature dependence of the oxygen vacancy diffusion coefficient (*D*_V_) of these oxides measured by Mizusaki et al. [71]. The oxygen vacancy diffusion coefficient of the perovskite-type oxides is close, both in absolute values and in activation energy. Thus, it is suggested that the diffusivity of oxygen vacancies is determined essentially by crystal structure.

## 6. High-temperature proton-conducting ceramics

**Figure 19.**
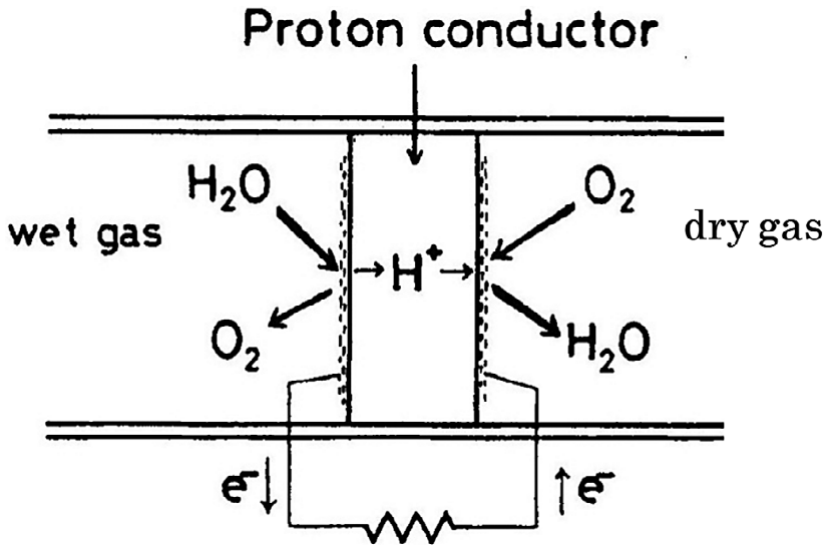
Schematic of steam concentration cell using a solid proton conductor as the electrolyte. Reprinted from [7] with permission from Elsevier.

Research on the high-temperature proton-conducting ceramics began in Japan. In 1981, Iwahara of Tottori University, who was a student of Professor Takahashi at Nagoya University, and his co-workers reported that strontium cerate based ceramics showed a relatively high proton conductivity at elevated temperatures [7]. A typical example of a proton conducting oxide of this class is SrCe_0.95_Y_0.05_O_3-*δ*_. This material is a substituted solid solution based on a perovskite-type oxide, in which 5 mol% of Ce are replaced with Y, and it exhibits protonic conduction in a hydrogen containing atmosphere at elevated temperature. The proton conductivity of this solid solution is approximately 10^–2^ S cm^–1^ at 900 °C in hydrogen atmosphere. Proton conduction in this oxide was verified by electrochemical hydrogen transport experiments under hydrogen or water vapor-containing atmosphere at elevated temperatures. A schematic of a steam concentration cell using a solid proton conductor is shown in Figure 19.

The proton conduction of this oxide is explained by the following mechanism. At higher oxygen partial pressure, this oxide exhibits a p-type (hole) conduction. When water vapor or hydrogen is introduced to the atmosphere, the concentration of holes decreases and that of protons increases according to the following equilibrium reactions:


(12)
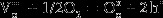




(13)
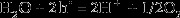




(14)
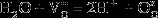




(15)
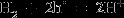



**Figure 20.**
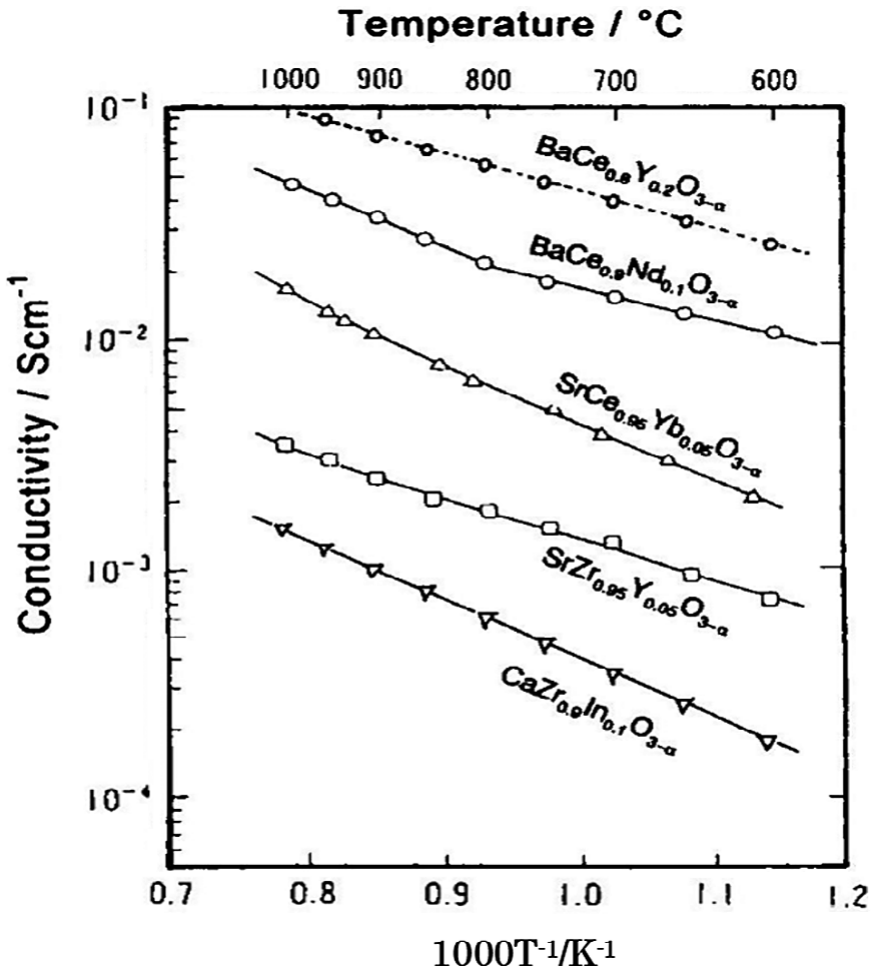
Conductivity of typical proton conducting perovskite-type oxide ceramics under a hydrogen gas atmosphere. Reprinted from [85] with permission from Elsevier.

They are written in the Kröger–Vink notation, where V_o_^••^ is oxygen vacancy, O_o_^x^ is oxygen in an oxygen site, and h• is a hole. The concentration of holes increases with oxygen partial pressure, while that of protons increases with water and hydrogen partial pressure. The proton transport number of SrCe_0.95_Y_0.05_O_3-*δ*_ was reported to be 0.99 at 700 °C with an H_2_O partial pressure of 0.002 MPa. The proton transport number decreased slightly with increasing temperature; that at 1000 °C was 0.84. High proton conductivity was observed in a series of doped alkali earth cerates ACe_1-*x*_M_*x*_O_3-*δ*_ (A = Sr, Ba, Ca; M = Y, Nd, Yb) [77–82]. Some doped zirconias based on CaZrO_3_, SrZrO_3_, and BaZrO_3_ [83,84] have also been confirmed to exhibit proton conduction. Figure 20 shows the temperature dependence of typical proton-conducing perovskite-type oxide ceramics under a hydrogen atmosphere. BaCeO_3_-based ceramics showed the highest conductivity among these oxides. However, the contribution of oxide ions to the conduction increases significantly as the temperature is raised.

**Figure 21.**
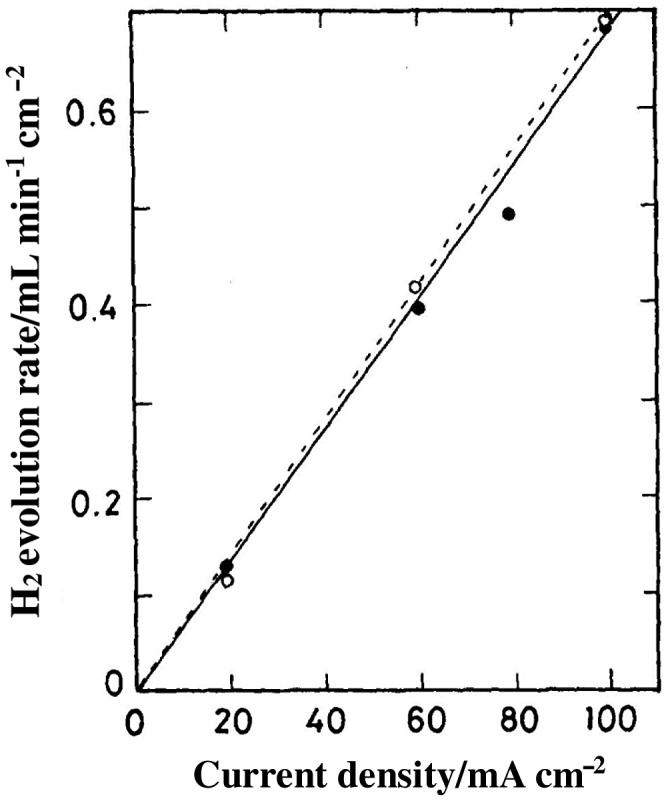
Plot of hydrogen evolution rate versus current density for H_2_,Pt/BaCe_1-*x*_Nd_*x*_O_3-δ_/Pt, Ar; ○: *x* = 0.10, ●: *x* = 0.05. Dashed line indicates the theoretical line. Replotted from [80].

Figure 21 shows the hydrogen evolution rate of a H_2_,Pt/BaCe_1-*x*_Nd_*x*_O_3-δ_/Pt,Ar cell as a function of current density at 800 °C. The evolution rate coincided with the theoretical rate calculated from Faraday’s law. This is evidence of proton conduction in these ceramics, because the proton formed at the anode (H_2_(g) → 2H^+^ + 2e^–^) must migrate across the oxide ceramic to discharge at the cathode (2H^+^ + 2e^–^ → H_2_(g)). Although the conductivity of SrCeO_3_-based ceramics is rather low, their proton transport number is higher than that of BaCeO_3_-based ceramics. The zirconia-based ceramics exhibit lower conductivity than the cerates, but are superior with respect to their chemical stability and mechanical strength.

Various applications can be devised using proton conducting ceramics [85,86]. Proton conducting ceramics can be used as hydrogen sensors for high-temperature industrial processes. Galvanic-cell-type hydrogen sensors, steam sensors, and hydrocarbon sensors that are usable at high temperature have been fabricated [87]. These sensors are essentially based on the principle of the hydrogen concentration cell using a proton conducting solid electrolyte.

Hydrogen sensors based on CaZr_0.9_In_0.1_O_3-*δ*_ ceramic have been commercialized by TYK, Japan [87] to measure the hydrogen partial activity in fused aluminum during the casting process. These sensors could also be applied to other fused metals such as zinc and copper. Iwahara has proposed other applications, such as a hydrogen fuel cell using SrCe_0.95_Yb_0.05_O_3-*δ*_ that exhibited stable operation at 800–1000 °C [80]. When the cell was discharged, water vapor was generated at the cathode at the same rate as the theoretical rate calculated from Faraday’s law, which indicates that the conduction in the electrolyte was protonic. When a hydrogen fuel cell is operated inversely, water vapor at the anode is decomposed to form protons that migrate to the cathode, where they discharge to produce hydrogen. This is a type of steam electrolyzer for hydrogen production. A bench-scale steam electrolyzer was fabricated using a ceramic tube of SrCe_0.95_Yb_0.05_O_3-δ_ electrolyte closed at one end. The electrolyzer was operated at 750 °C, and produced pure and very dry hydrogen (dew point < −30 °C) that could be extracted at a rate of 3 L h^–1^ [85].

## 7. Highly oxide-ion and hydride-ion conducting solid conductors

Since Nernst reported the oxide-ion conduction of doped zirconia in 1897 [88], the zirconia family of oxide-ion conductors has been extensively studied by many research groups. The candidate electrolytes can be divided into two major structures; fluorite and perovskite. Both ceria and high temperature-stabilized zirconia take a fluorite structure, and lanthanum gallates have the perovskite structure. The fluorite structure is a face-centered cubic arrangement of cations with anions occupying all the tetrahedral sites, which leads to a large number of octahedral voids. This arrangement is rather open and, thus, rapid ion diffusion may be expected. At high temperature, zirconia has the fluorite structure, and the high-temperature phase can be stabilized at lower temperatures by the addition of divalent or trivalent cations such as Ca^2+^ or Y^3+^ [88]. The dissolution of yttria into the fluorite phase of ZrO_2_ can be expressed by the following defect equation in the Kröger–Vink notation:(16)
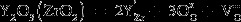



**Figure 22.**
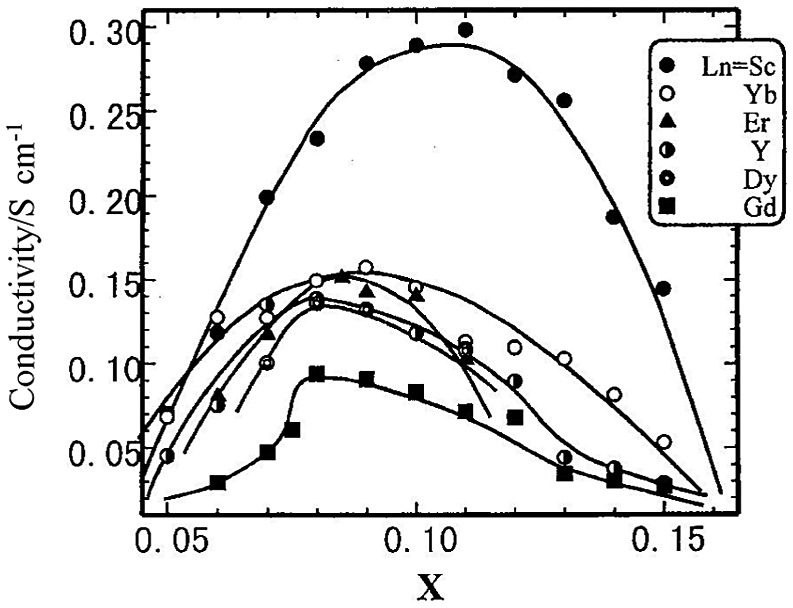
Compositional dependence of the electrical conductivity of the ZrO_2-*x*_Ln_2_O_3_ system at 1000 °C. Reprinted from [90] with permission from Elsevier.

Each additional yttria molecule creates one oxygen vacancy. The conductivity of doped zirconia varies as a function of dopant concentration and has a maximum at or near the minimum quantity of dopant required to fully stabilize the cubic fluorite phase [89]. Arachi et al. [90] analyzed the effect of the dopant on the conductivity of ZrO_2_–Ln_2_O_3_ (Ln = Sc, Yb, Er, Y, Dy, or Eu). The dependence of the conductivity on the dopant concentration at 1000 °C is shown in Figure 22. Doping with Sc^3+^, which has the closest ionic radius to the host ion Zr^4+^, results in the highest conductivity of approximately 0.3 S cm^–1^ at 1000 °C. However, this system has been reported to exhibit an aging effect upon annealing at high temperature [91]. The change in the electrical conductivity of the ZrO_2_–Sc_2_O_3_ system (ScSZ) after an annealing period at 1000 °C was examined by Yamamoto et al. [92]. The change in conductivity with the annealing period was found to be dependent on the dopant content. ScSZ with 8 mol% Sc_2_O_3_ exhibited a significant aging effect upon annealing at 1000 °C. The conductivity of 0.3 S cm^–1^ for the as-sintered ScSZ at 1000 °C decreased to 0.12 S cm^–1^ after aging for 1000 h. In contrast, ScSZ with 11 mol% Sc_2_O_3_ (11ScSZ) showed no aging effect, even after annealing at 1000 °C for 6000 h. However, 11ScSZ shows a phase transition at 600 °C with a small volume change. Mizutani et al. reported that the cubic phase of 11ScSZ is stabilized at room temperature by the addition of small amounts of Al_2_O_3_ [93]. The electrical conductivity of 11ScSz-1 wt%Al_2_O_3_ was found to be 0.2 S cm^–1^ at 1000 °C, which is slightly lower than that of 11ScSZ, but this composition shows no phase transition. This type of scandia-doped zirconia oxide is now widely used as an electrolyte for SOFCs.

**Figure 23.**
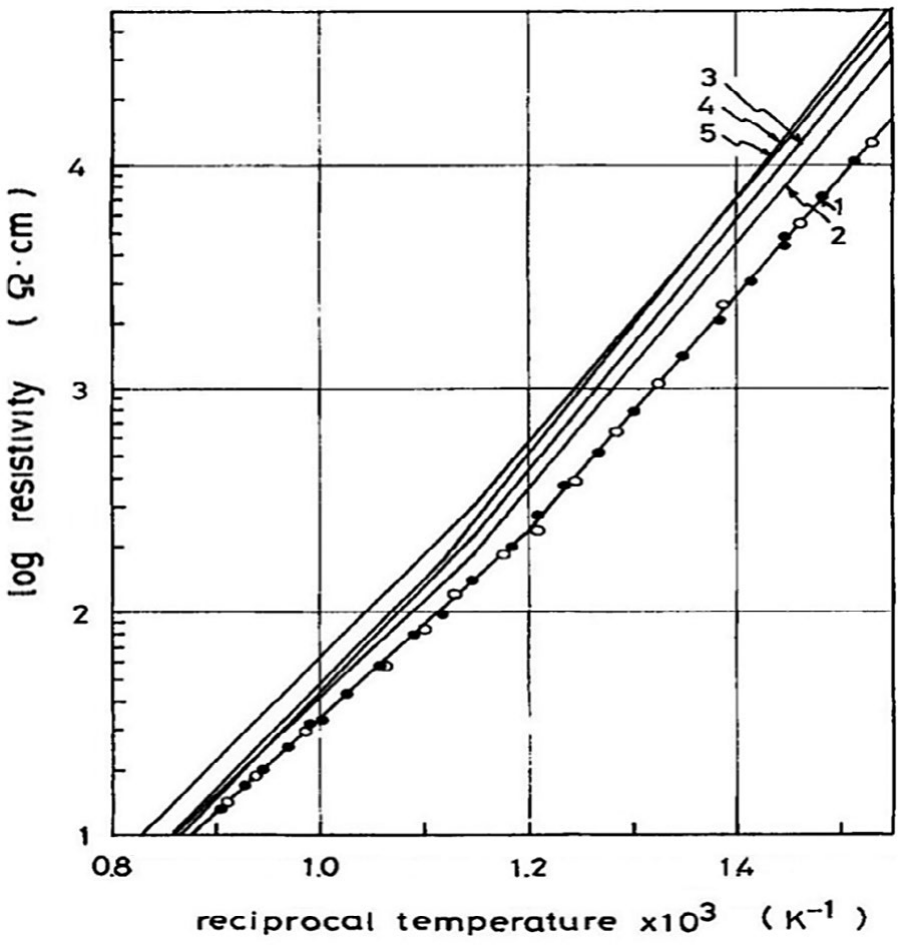
Arrhenius plot of resistivity for Ce_0.7_Ln_0.3_O_1.85_. Lines 1–5 are for Ln = Gd, Dy, Ho, Er, and Yb, respectively. Reprinted with permission from *J. Electrochem. Soc.*, **122**, 142 (1975). Copyright 1975, The Electrochemical Society [94].

**Figure 24.**
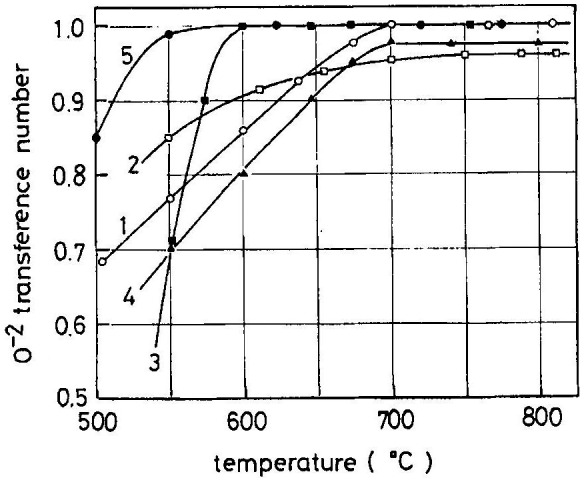
Oxide-ion transport number for Ce_1.7_Ln_0.3_O_1.85_. Curves 1−5 are for Ln = Nd, Sm, Gd, Dy and Er, respectively. Reprinted with permission from *J. Electrochem. Soc.*, **122**, 142 (1975). Copyright 1975, The Electrochemical Society [94].

The other reported fluorite-type oxide-ion conductors are based on CeO_2_ [88]. However, systematic research on this system was lacking before the study by Kudo and Obayashi [94] in 1975. They systematically studied the oxide-ion conductivity and the transport number of fluorite-type Ce_1-*x*_Ln_*x*_O_2-*x*/2_ (Ln = lanthanide element) as a function of the dopant content and oxygen partial pressure. The maximum conductivity was found at around *x* = 0.3 with a lattice constant of 0.53 to 0.54 nm. Figure 23 shows temperature dependence of the resistivity in the Ce_0.7_Ln_0.3_O_1.85_ system. The conductivity of Ce_0.7_Gd_0.3_O_1.85_ at 750 °C is 7 × 10^–2^ S cm^–1^, which is several times higher than that of zirconia-based oxide-ion conductors. However, at 1000 °C, its conductivity is comparable to that of zirconia-based oxide-ion conductors. The ceria-based oxide-ion conductors exhibit high conductivity at lower temperatures and have applied as the electrolyte for intermediate temperature SOFCs. Ceria-based oxide-ion conductors were found to show electron hole conduction under reduced atmosphere [88]. Kudo and Obayashi measured the ionic transport number of the ceria-based oxide-ion conductors using an oxygen concentration cell. Figure 24 shows the oxide-ion transport numbers for Ce_0.7_Ln_0.3_O_1.85_. The transport number increases with temperature and was less than 0.95 for Ce_0.7_Ln_0.3_O_1.85_ (Ln = Sm or Dy), whereas that of the compounds containing Nd, Gd and Er was unity at high temperatures.

**Figure 25.**
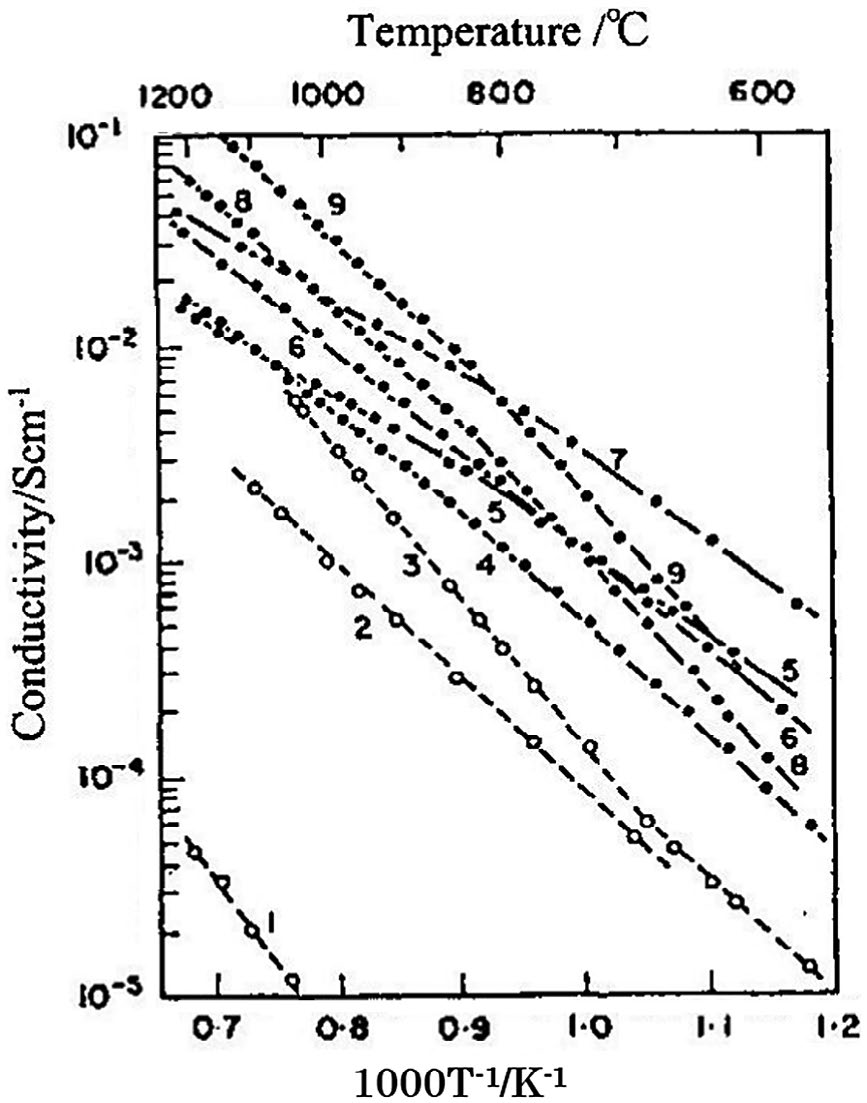
Arrhenius plots of the oxide-ion conductivity of the perovskite-type oxides measured in air; 1: LaAlO_3_, 2: CaTiO_3_, 3: SrTiO_3_, 4: La_0.7_Ca_0.3_AlO_3_, 5: La_0.9_Ba_0.1_AlO_3_, 6: SrTi_0.9_Al_0.1_O_3_, 7: CaTi_0.95_Mg_0.05_O_3_, 8: CaTi_0.5_Al_0.5_O_3_, 9: CaTi_0.9_Al_0.1_O_3_. Reprinted from [73] with permission from Elsevier.

In addition to the fluorite structure oxide-ion conductors, some perovskite-type oxides exhibit high ion conductivity. In 1971, Takahashi and Iwahara reported the electrical conductivities of some perovskite-type oxides [73]. Figure 25 shows the temperature dependence of many perovskite-type oxides. The highest conductivity was found for Al- or Mg-doped CaTiO_3._ However, the CaTiO_3_-based oxides had low ionic transport numbers at high temperature and in reduced atmosphere.

**Figure 26.**
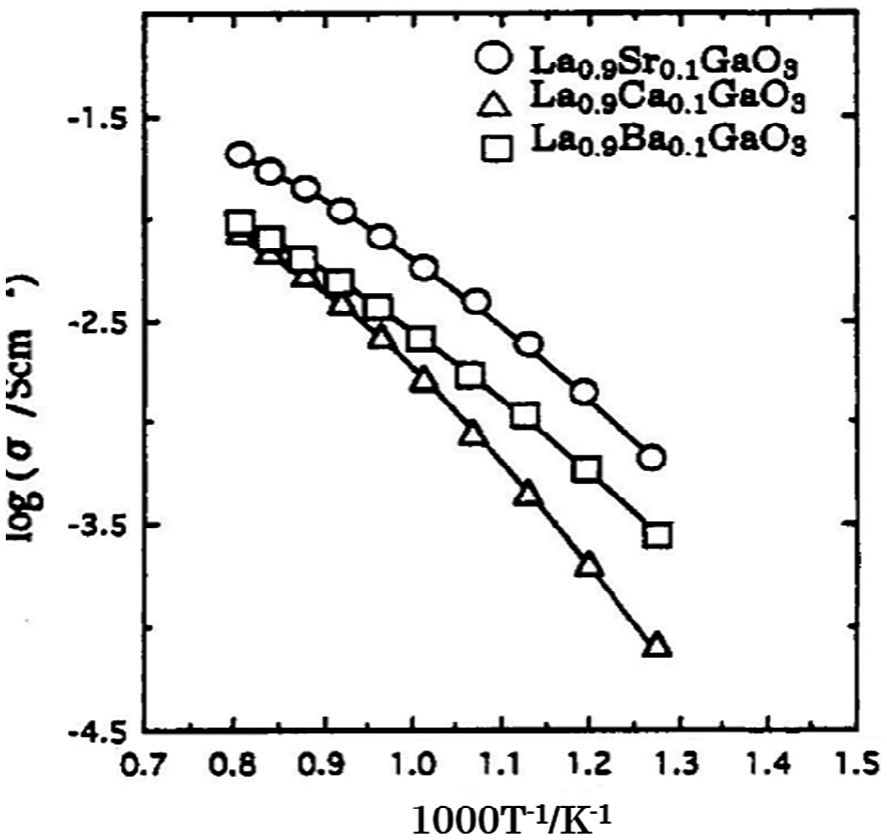
Effect of alkaline earth cation doping at the La sites in LaGaO_3_ on the electrical conductivity of La_1-*x*_M_*x*_GaO_3_ for M = Ca, Sr, Ba (P_O2_ = 10^–5^ atm). Reprinted from [8] with permission. Copyright 1994 American Chemical Society.

The electrical conductivities reported by Takahashi and Iwahara for the perovskite-type oxides were lower than those of the fluorite-type oxides. After the pioneering work by Takahashi and Iwahara, some perovskite-type oxides were found to have high oxide-ion conductivity. Cook et al. [95] reported a high electrical conductivity for BaTh_0.9_Gd_0.1_O_3_, 8.7 × 10^–2^ at 550 °C. In 1994, Ishihara et al. [8] and Feng and Goodenough [96] reported high oxide-ion conductivities for doped perovskite-type oxides of LaGaO_3_. In particular, Ishihara et al. reported a high conductivity for La_0.9_Sr_0.1_Ga_0.9_Mg_0.1_O_3_. Figure 26 shows the effect of the addition of alkaline earth cations to the La site in LaGaO_3_ on its electrical conductivity. The electrical conductivity of LaGaO_3_ was dependent on the added alkaline earth cation and increased in the order Ca < Ba < Sr. The electrical conductivity increased with the amount of Sr additive and attained a maximum at *x* = 0.1. Impurity phases of SrGaO_3_ and LaSrO_3_ were detected above *x* = 0.1.

**Figure 27.**
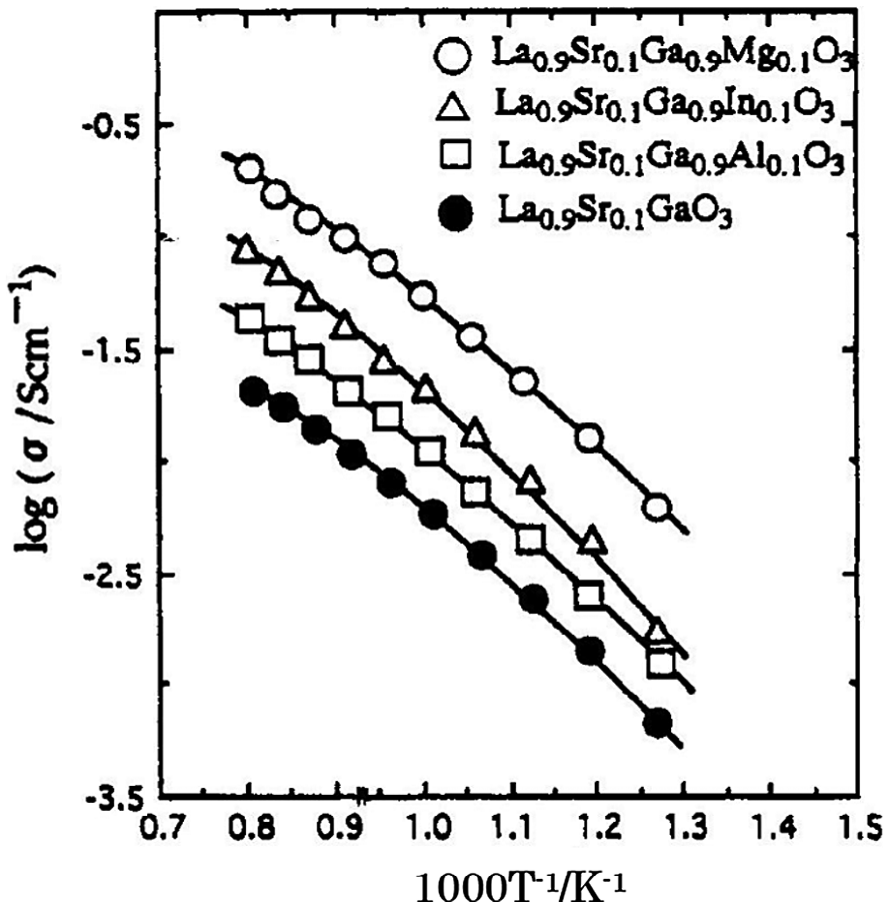
Effect of doping of various cations into the Ga sites in LaGaO_3_ on the electrical conductivity of La_0.9_Sr_0.1_Ga_0.9_M_0.1_O_3_ for M = Al, In, Mg, (P_O2_ = 10^–5^ atm.). Reprinted from [8] with permission. Copyright 1994 American Chemical Society.

**Figure 28.**
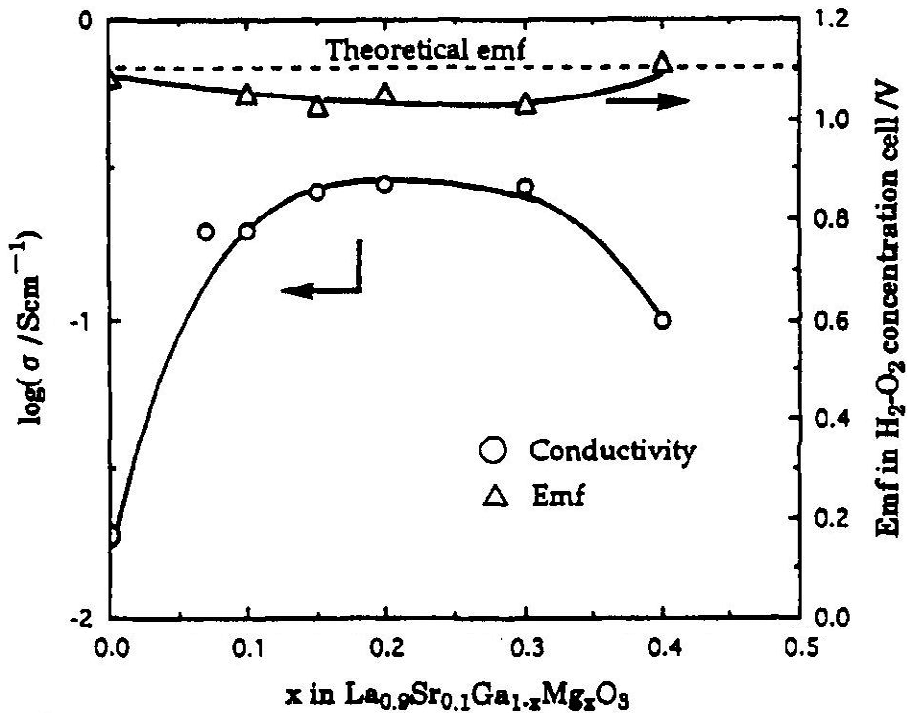
Effect of amount of Mg substitution on the electrical conductivity of La_0.9_Sr_0.1_Ga_1-*x*_Mg_*x*_O_3_ at 1223 K (P_O2_ = 10^–5^ atm.) and emf of an H_2_-O_2_ concentration cell based on La_0.9_Sr_0.1_Ga_1-*x*_Mg_*x*_O_3_ at 1273 K. Reprinted from [8] with permission. Copyright 1994 American Chemical Society.

The effect of additives at the Ga site of La_0.9_Sr_0.1_GaO_3_ on the electrical conductivity was also studied. The results are shown in Figure 27. The highest electrical conductivity was found for La_0.9_Sr_0.1_Ga_0.9_Mg_0.1_O_3_, as high as 0.1 S cm^–1^ at 800 °C. The ionic transport number of La_0.9_Sr_0.1_Ga_1-*x*_Mg_*x*_O_3_ was examined using a H_2_-O_2_ concentration cell. Figure 28 shows the effect of the amount of Mg substitution on the electrical conductivity of La_0.9_Sr_0.1_Ga_1-*x*_Mg_*x*_O_3_ at 1223 K and E for a H_2_-O_2_ concentration cell with La_0.9_Sr_0.1_Ga_1-*x*_Mg_*x*_O_3_. The electrical conductivity increased with the Mg content and a maximum was observed for La_0.9_Sr_0.1_Ga_1-*x*_Mg_*x*_O_3_ at *x* = 0.2. The concentration cell had an almost theoretical emf at P_O2_ = 10^–5^ atm. The electrical conductivity did not change within an oxygen partial pressure range of 1 to 10^–21^ atm. at 1123 K, which suggests that the Mg-doped La_0.9_Sr_0.1_Ga_1-*x*_Mg_*x*_O_3_ perovskite-type oxide is almost a pure oxide-ion conductor over a wide range of oxygen partial pressures. The advantage of the LaGaO_3_-based oxide ion conductors is a high conductivity in the lower temperature range. Ishihara et al. have extensively studied intermediate temperature SOFCs using this type of oxide-ion conductor. SOFCs are typically operated at around 1000 °C because of the conductivity limitation of the conventional Y_2_O_3_ stabilized ZrO_2_ (YSZ) oxide-ion conductor. SOFCs containing LaGaO_3_-based oxides exhibit a high power density of 0.5 W cm^–2^ at 700 °C, which is comparable to that of SOFCs with YSZ electrolyte at 1000 °C [97].

**Figure 29.**
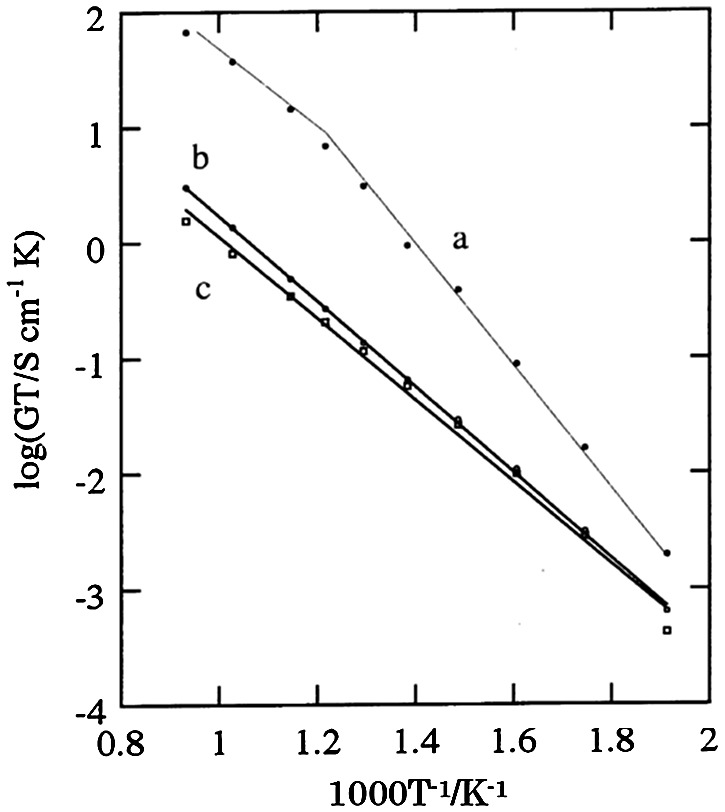
Temperature dependence of electrical conductivity for (a) Y_2_O_3_ stabilized ZrO_2_, (b) La_10_(SiO_4_)_6_O_3_ and (c) Nd_10_(SiO_4_)_6_O_3_. Reproduced with permission from [98].

In 1995, a new class of highly oxide-ion conducting solid electrolytes was reported by Nakayama et al. [98]. They investigated the electrical conductivity of lanthanide silicate oxyapatites of Ln_10_(SiO_4_)_6_O_3_ (Ln = La, Nd, Sm, Gd, and Dy) in composition and found the highest conductivity of 2.3 × 10^–4^ S cm^–1^ at 500 °C for Nd_10_(SiO_4_)_6_O_3_. The temperature dependence of the electrical conductivity of Nd_10_(SiO_4_)_6_O_3_ and La_10_(SiO_4_)_6_O_3_ is shown in Figure 29. The oxide-ion transport number of Nd_10_(SiO_4_)_6_O_3_ was estimated to be around unity using an oxygen gas concentration cell, O_2_–Ar/ Nd_10_(SiO_4_)_6_O_3_/air. Many studies related to their crystal structure and conductivity have been published since the discovery of the lanthanum silicate oxyapatites by Nakayama et al. Yoshioka and Tanase [99] reported a high conductivity of 4.5 × 10^–3^ S cm^–1^ at 500 °C for La_9.6_Si_5.7_Mg_0.3_O_26,1_, five times higher than that for Y_2_O_3_ stabilized ZrO_2_ [88] and comparable to that for the perovskite oxide La_0.9_Sr_0.1_Ga_0.9_Mg_0.1_O_3_ [8]. The crystal structure of the oxyapatites has been extensively studied; the diffusion path was proposed for La_9.33_Si_6_O_26_ and La_8_Sr_2_Si_6_O_26_ by Sansom et al. [100] and for La_9.69_Si_5.7_Mg_0.3_O_26,24_ by Ali et al. [101] using neutron diffraction analysis. These results suggested that La_9.33_Si_6_O_26_ and La_9.69_Si_5.7_Mg_0.3_O_26,24_ have oxide-ion lattice vacancies and oxide ions migrate via a vacancy mechanism. As shown by the electrical conductivity data for lanthanum silicate oxyapatites by Kobayashi and Sakka [102], the conductivity was dependent on the preparation method because of high grain boundary resistances and impurity phases. La_8_Sr_2_Si_6_O_26_ has no oxide-ion vacancies and its electrical conductivity is low [100]. To obtain a high oxide-ion conductivity, oxyapatites with high oxide-ion vacancy should be prepared

**Figure 30.**
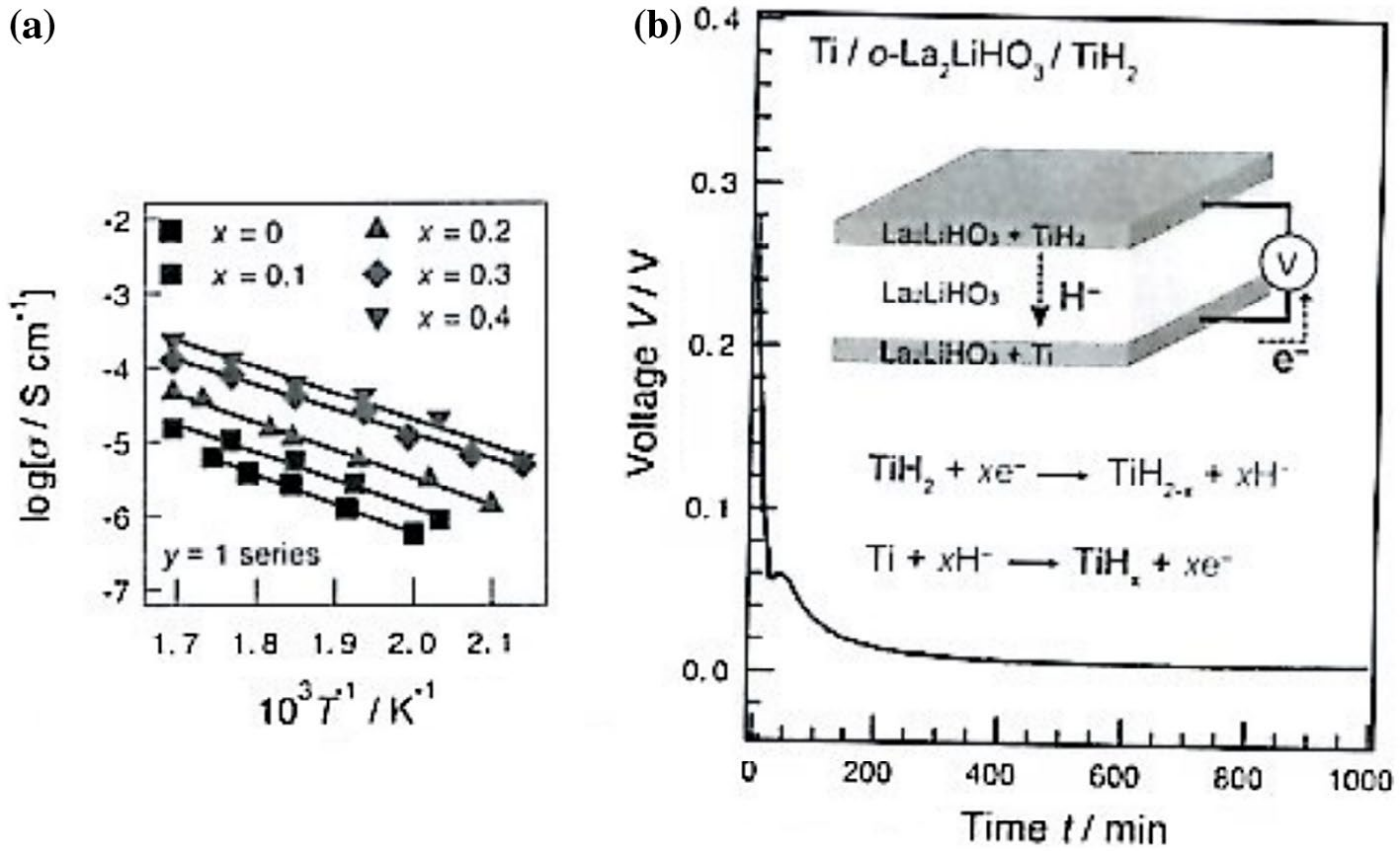
(A) Temperature dependence of the ionic conductivity of La_1-*x*_Sr_1+*x*_LiH_2-*x*_O_2_; and (B) discharge curve for the Ti/orthorhombic La_2_LiHO_3_/TiH_2_ cell at 300 °C. From [104]. Reprinted with permission from AAAS.

Recently, Irvine and co-workers reported a high H^–^ ion conduction in BaH_2_, the electrical conductivity of which was 0.2 S cm^–1^ at 630 °C [103]. Although some conductivity studies have been reported for related materials, the nature of the charge carriers has not yet been determined. More recently, Kanno and co-workers found that a series of K_2_NiF_4_-type oxyhydrides, La_2-*x*-y_Sr_*x*+y_LiH_1-*x*+y_O_3-y_, show hydride-ion conductivity [104]. Hydride-ion solid conductors are attractive as an electrolyte for high energy density batteries, because of their strong reducing properties and standard potential of H_2_/H^–^ (−2.3 V), which is close to that of Mg^2+^/Mg (−2.4 V). Utilization of alkaline earth hydrides as a battery electrolyte is difficult because of their structural inflexibility. Figure 30 shows the temperature dependence of the electrical conductivity of La_1-*x*_Sr_1+*x*_LiH_2-*x*_O_2_. An electrical conductivity of 2.1 × 10^–4^ S cm^–1^ was observed at 317 °C for La_0.6_Sr_1.4_LiH_1.6_O_2_. To identify the nature of the charge carriers, the electron (and hole) conductivity was measured by the Hebb-Wagner polarization method [105] using an asymmetric (-)Pd/La_0.6_Sr_1.4_LiH_1.6_O_2_/Mo(+) cell at 207 and 317 °C. The total conductivity (electron + hole) at 207 and 317 °C was 2.9 × 10^–8^ and 4.1 × 10^–7^ S cm^–1^, respectively.

It is evident that La_0.6_Sr_1.4_LiH_1.6_O_2_ is a pure ionic conductor. An all-solid-state Ti/o-La_2_LiHO_3_/TiH_2_ cell was constructed to confirm the ion carrier in orthorhombic La_2_LiHO_3_. Figure 30(B) shows the discharge curve at a constant current of 0.5 μA and 300 °C. The cell showed an initial open circuit voltage of 0.28 V, which is consistent with the theoretical value calculated from the standard Gibbs energy of formation of TiH_2_. The cell reaction products were analyzed by synchrotron XRD. Phase changes detected for the cathode and anode materials were consistent with those expected from the Ti–H phase diagram. From these results, Kanno et al. concluded that La_1-*x*_Sr_1+*x*_LiH_2-*x*_O_2_ is a pure hydride-ion conductor. Yamaguchi predicted that this result by Kanno and co-workers is just the beginning of a new field of material science of H^–^ conductivity in oxyhydride systems that will require further elaboration of the underlying mechanism, as well as potential application of the extremely good reducing ability of H^–^ ions in chemical synthesis [106].

## 8. Highly lithium-ion conducting solid electrolytes

**Figure 31.**
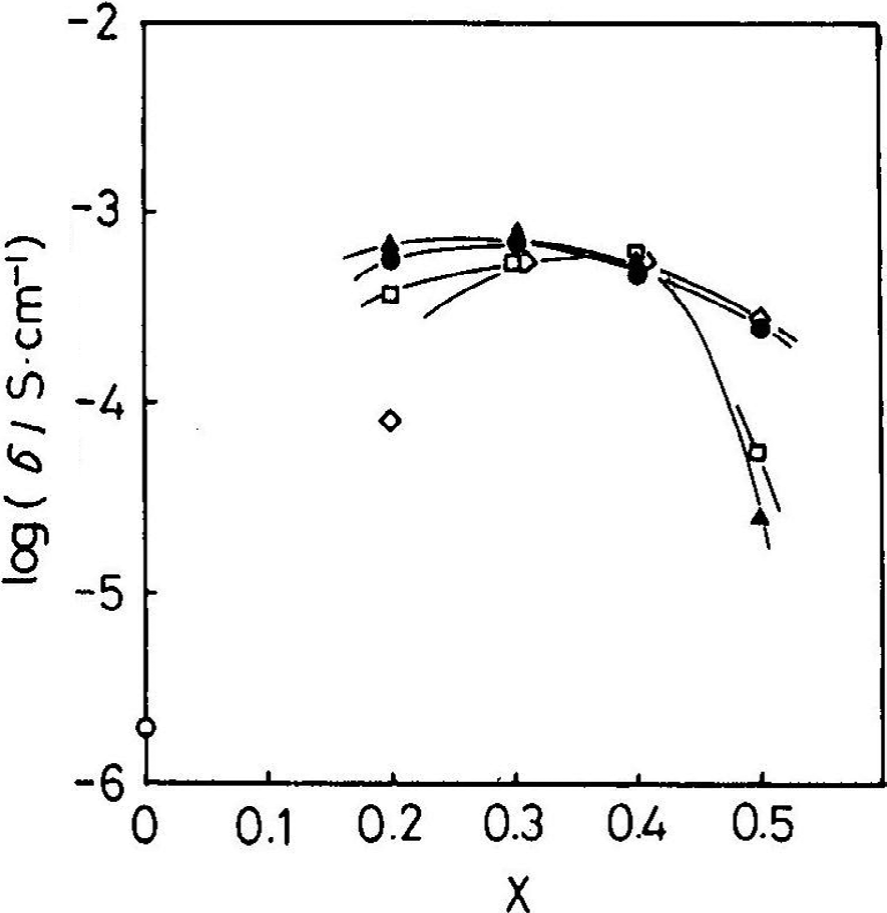
Conductivity measured at 25 °C as a function of *x* in Li_1+*x*_M_*x*_Ti_2-*x*_(PO_4_)_3_ ○: LiTi_2_(PO_4_)_3,_ ●: Li_1+*x*_Al_*x*_Ti_2-*x*_(PO_4_)_3_, ▲: Li_1+*x*_Sc_*x*_Ti_2-*x*_(PO_4_)_3_, □: Li_1+*x*_Y_*x*_Ti_2-*x*_(PO_4_)_3_, ◇: Li_1+*x*_La_*x*_Ti_2-*x*_(PO_4_)_3_. Reprinted with permission from *J. Electrochem. Soc.*, **136**, 590 (1989). Copyright 1989, The Electrochemical Society [107].

Many new types of highly lithium-ion conducting solid electrolytes have been reported by Japanese researchers. In 1989, Aono et al*.* [107] reported a highly lithium-ion conducting solid electrolyte of composition Li_1+*x*_M_*x*_Ti_2-*x*_(PO_4_)_3_ (M = Al, Sc, Y, and La). This system has the NASICON-type structure. Figure 31 shows the conductivity of Li_1+*x*_M_*x*_Ti_2-*x*_(PO_4_)_3_ at 25 °C as a function of *x*, in which the highest conductivity was observed at *x* = 0.3 for all systems examined. The conductivity of Li_1.3_Al_0.3_Ti_1.7_(PO_4_)_3_ at 25 °C almost attains the values previously reported for the highly lithium-ion conducting LISICON-type solid electrolytes with composition Li_2+2*x*_Zn_1-*x*_GeO_4_ [108] and for the layered-structure material Li_3_ N [109]. LISICON-type conductors and Li_3_ N are unstable in ambient atmosphere, but the NASICON-type lithium conducting solid electrolytes are stable both in ambient atmosphere and in aqueous solution with a high content of LiCl and LiNO_3_ [110]. This type of lithium-ion solid conductors has been used as a protective layer for the water-stable lithium electrode of aqueous lithium-air batteries by Visco et al. [111] and Imanishi et al. [112].

**Figure 32.**
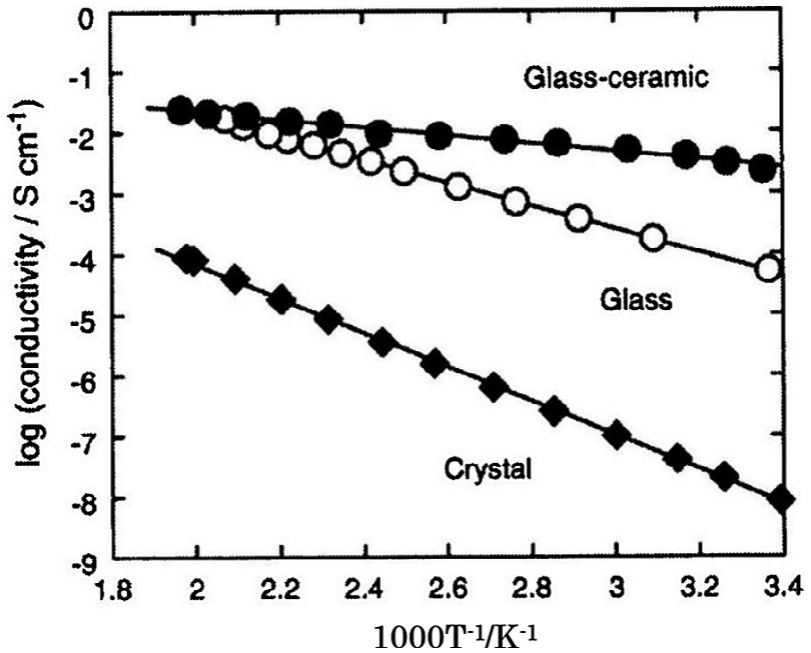
Temperature dependence of electrical conductivity of 70Li_2_S-30P_2_S_5_. Reprinted from [113] with permission from Elsevier.

The crystallization of glassy materials is generally known to lower conductivity [113]. However, the enhancement of conductivity by careful heat-treatment was reported for Li_2_S-P_2_S_5_ glasses by Tatsumisago and co-workers in 2001 [114]. Figure 32 shows the temperature dependence of the electrical conductivity of 70Li_2_S-30P_2_S_5_ samples prepared by mechanical milling and solid-state reaction. The crystallized sample was obtained by solid-state reaction of Li_2_S-P_2_S_5_ and glass-ceramic by crystallization of the glass at 360 °C. The room temperature conductivity of the glass was 5.4 × 10^–5^ S cm^–1^, while that of the glass-ceramic was 3.2 × 10^–3^ S cm^–1^. The activation energy for conduction of the glass-ceramic was as low as 13 kJ mol^–1^, which is approximately one-third of that of the glass. This conductivity enhancement by crystallization of the glass is explained by the precipitation of a highly lithium-ion conducting crystal phase of Li_2_S-P_2_S_5_ [115]. A new crystalline phase was observed in the glass-ceramic heated at 240 and 360 °C that was not obtained in solid-state reactions. The crystalline structure of the new phase Li_7_P_3_S_11_ was determined from synchrotron XRD data [116]. The high conductivity of the glass-ceramic heat-treated at 360 °C was caused by both the precipitation of the Li_7_P_3_S_11_ phase and the increase in the crystallinity of the phase. The lower conductivity Li_4_P_2_S_6_ and Li_3.55_P_0.89_S_4_ crystals were mainly present in the sample obtained by solid-state reaction. Thus, the highly conducting L_7_P_3_S_11_ crystals precipitate as a metastable phase when the glass is crystalized. The metastable phase is responsible for the high conductivity and low activation energy for conduction of 70Li_2_S-30P_2_S_5_. Tatsumisago and Hayashi have extensively examined all-solid-state batteries containing this highly lithium-ion conducting glass ceramic compound [117]. Figure 31 shows some results for all-solid-state batteries with a Li-In anode, a highly lithium-ion conducting glass ceramic electrolyte, and a sulfur cathode. Excellent charge–discharge performance was obtained at room temperature and a current density of 0.046 mA cm^–2^. The all-solid-state batteries with sulfur exhibited a much larger capacity than that of the conventional lithium-ion battery with LiCoO_2_, greater than 1000 mAh g^–1^. In the case of Li-In/S batteries with S-C composite, this large capacity was retained after many charge–discharge cycles [118].

**Figure 33.**
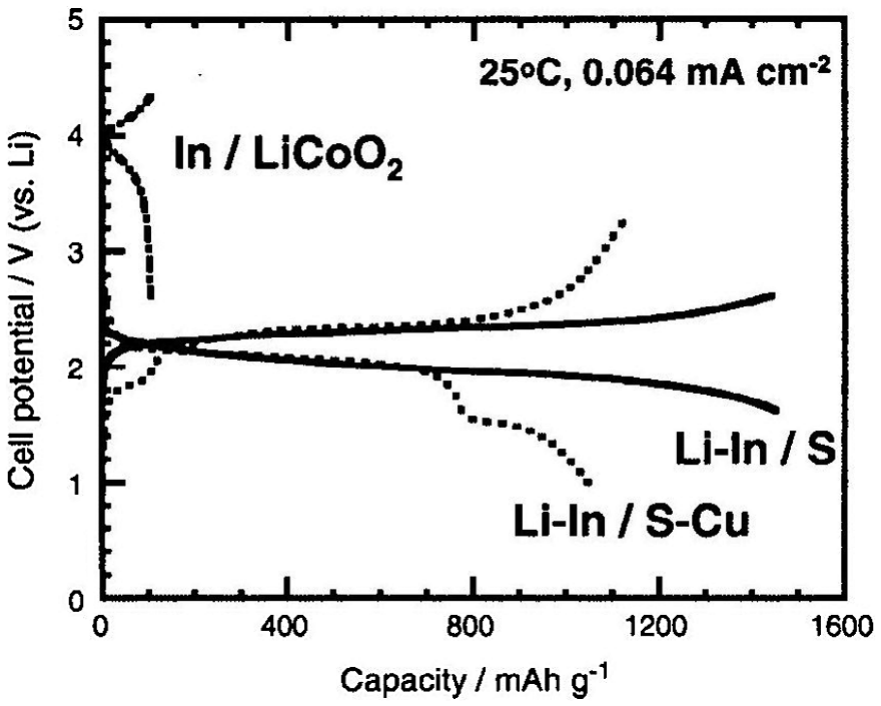
Charge-discharge curves for all-solid-state Li-In/80Li_2_S-20P_2_S_5_ glass ceramic/S-C (or S-Cu, LiCoO_2_) cell. Reprinted from [117] with permission from Elsevier.

Takada and coworkers [119,120] reported that the low capacity of a cell with a LiCoO_2_ cathode could be explained by a space charge layer formed between the electrolyte and LiCoO_2_, which resulted in the formation of a large interface resistance. They improved the interface resistance by adding an interlayer of Li_4_Ti_5_O_12_ or LiNbO_3_ between the electrolyte and LiCoO_2_

**Figure 34.**
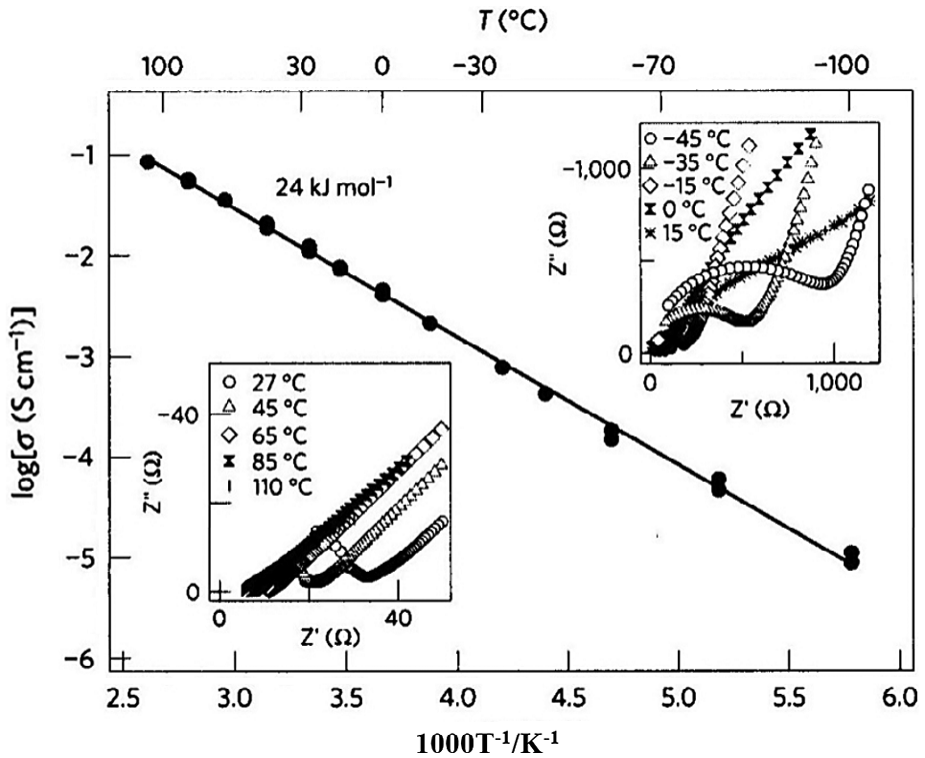
Lithium ion conductivity of Li_10_GeP_2_S_12_. Reprinted by permission from Macmillan Publishers Ltd [9], copyright 2011.

A new thio-LISICON crystalline material family was found in the Li_2_S-GeS_2_-P_2_S_5_ system by Kanno and Murayama in 2001 [121]. The solid solution member *x* = 0.75 in Li_4-*x*_Ge_1-*x*_P_*x*_S_4_ showed the highest conductivity of 2.2 × 10^–3^ S cm^–1^ at 25 °C, together with negligible electronic conductivity, high electrochemical stability, no reaction of lithium metal, and no phase transition up to 500 °C. The thio-LISICON has a similar structure to that of the Li_14_Zn(GeO_4_)_4_ LISICON [122]. Kanno and co-workers [9] reported on the discovery of a fast lithium-ion conductor of composition Li_10_GeP_2_S_12_, the conductivity of which is as high as 0.012 S cm^–1^ at room temperature. The lithium ion conductivity of this solid electrolyte is higher than those of the conventional non-aqueous electrolytes, and its room temperature lithium ion conductivity is the highest reported to date. Li_10_GeP_2_S_12_ was synthesized by reacting stoichiometric quantities of Li_2_S, GeS_2_, and P_2_S_5_ at 550 °C in an evacuated quartz tube. The XRD pattern of the reaction product indicated a new phase with a structure that differed from those of previously reported lithium-ion conductors such as thio-LISICON Li_7_PS_5._ The composition and the structure of Li_10_GeP_2_S_12_ were determined by synchrotron XRD and neutron diffraction measurements. Figure 34 shows the conductivity measurement results for Li_10_GeP_2_S_12_._._ The activation energy for conduction was found to be 24 kJ mol^–1^.

**Figure 35.**
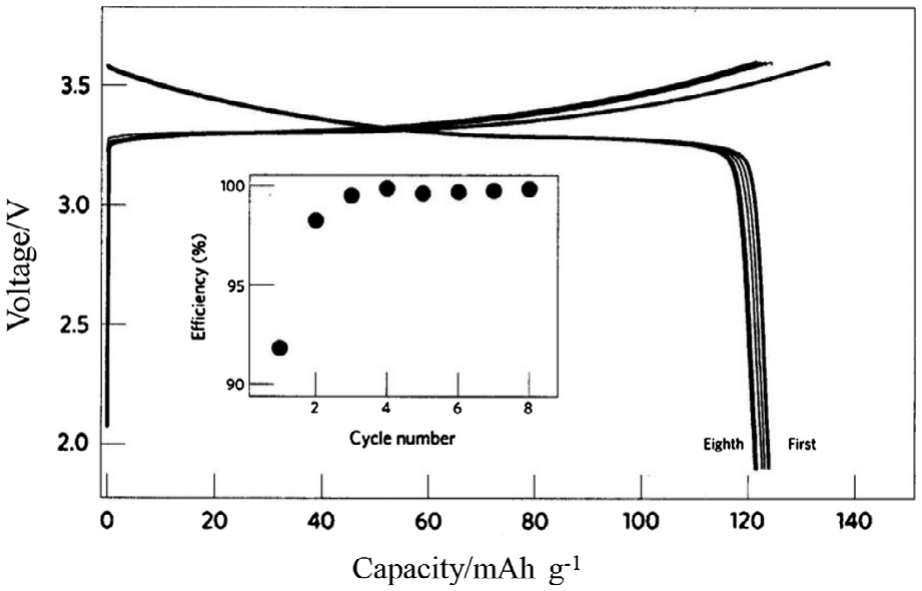
Charge-discharge curves of an all-solid-state battery consisting of a LiCoO_2_ cathode, a Li_10_GeP_2_S_12_ electrolyte, and an In metal anode. Reprinted by permission from Macmillan Publishers Ltd [9], copyright 2011.

The electrochemical stability of Li_10_GeP_2_S_12_ was examined by cyclic voltammetry of a Li/Li_10_GeP_2_S_12_/Au cell with a lithium reference electrode. No significant current due to electrolyte decomposition was detected up to 5 V. The electronic conductivity at 25 °C was measured by the Hebb-Wagner polarization method using a Li/Li_10_GeP_2_S_12_/Au cell. The total electronic conductivity (electron and hole) was found to be 5.7 × 10^–9^ S cm^–1^. The new highly lithium-ion conducting solid electrolyte was identified as well suited for all-solid-state lithium batteries. Figure 35 shows the charge–discharge curves of a battery that consisted of a LiCoO_2_ cathode, a Li_10_GeP_2_S_12_ electrolyte, and an In metal anode at a current density of 14 mA g^–1^. The battery exhibited a discharge capacity of 120 mAh g^–1^ and an excellent charge and discharge efficiency of approximately 100% after the second cycle, which demonstrated Li_10_GeP_2_S_12_ to be applicable as a practical electrolyte for all-solid-state batteries. More recently, Kanno and co-workers [123] found a new lithium-ion conducting solid electrolyte, Li_9.54_Si_1.74_P_1.44_S_11.7_Cl_0.3_, the lithium-ion conductivity of which is 2.5 × 10^–2^ S. This conductivity value is about double those of Li_10_GeP_2_S_12_ and conventional non-aqueous lithium conducting liquid electrolytes. The crystal structure of the new compound was analyzed by high power neutron diffraction analysis

## 9. Applications of solid ionic materials in Japan

Solid ionic materials have been mainly used in two industrial fields, sensors [124] and batteries [125]. Most cars have oxygen sensors to control the exhaust gas from their engines. These oxygen sensors typically contain the stabilized zirconia oxide-ion conducting solid electrolyte that was used as a heat element in 1897 by Nernst. At present, 67% of the zirconia-type oxygen sensors in all cars are supplied by Japanese companies.

In the battery industry, many types of solid ionic materials have been used. The largest commercial application for solid ionic materials is lithium-ion batteries, which were first commercialized by Sony, Japan, in 1990. Lithium-ion and electron mixed conductors such as LiCoO_2_ and LiC_6_ have been widely used as cathode and anode materials, for which high lithium-ion diffusion is desirable to achieve a high power density. Small-sized lithium-ion batteries have a large market in portable electronic equipment such as mobile phones. More recently, lithium-ion batteries have been used in electric and hybrid vehicles by Japanese motor companies, and are also planned for use in the storage of electricity from solar cells and wind power generators. Thus, the lithium-ion battery market is expected to grow rapidly in the next ten years, both in Japan and globally.

SOFCs also consist of solid state ionic materials; an oxide-ion conductor as the electrolyte and a perovskite-type oxide for the air electrode. Many Japanese companies have been developing SOFCs for many years, and a 0.7 kW-type SOFC system had been commercialized. The flat tubular-type SOFC stack was designed by Kyocera, Japan. Its energy efficiency for electricity is 46.5% lower heating value (LHV), and its total efficiency, including hot water generation, is 80% (LHV). The price of the unit is approximately US$26,000. At present, the cost of such units remains expensive and a government support through subsidies would increase the uptake by private households.

**Figure 36.**
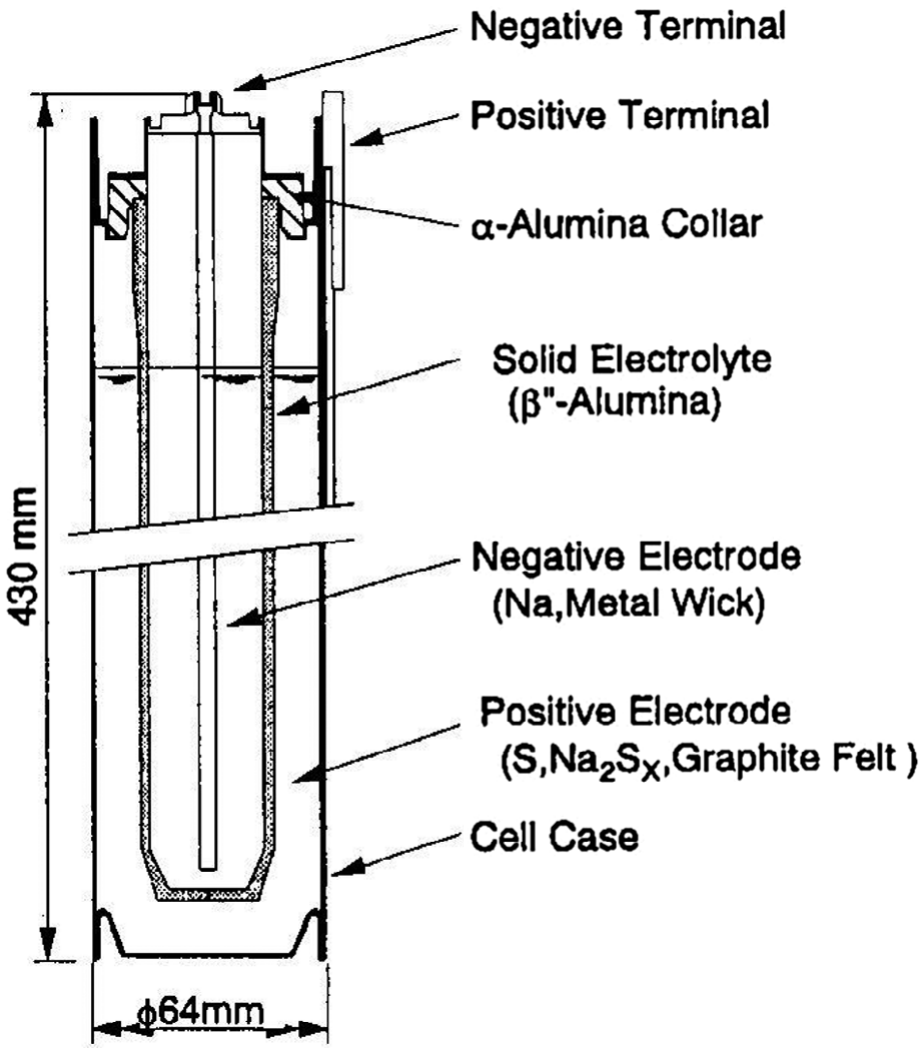
Cross-sectional view of a NAS battery. Reprinted from [130] with permission from Elsevier.

The most successful application of solid state ionic materials in Japan is sodium-sulfur batteries (NAS batteries). NAS batteries consist of a sodium anode, a β-alumina sodium-ion conducting solid electrolyte, and a sulfur cathode. This battery system was proposed by Ford Motors in the 1960s [126]. The key material is the β-alumina sodium-ion conducting solid electrolyte. Yao and Kummer [127] reported that β-alumina exhibits rapid sodium-ion diffusion, and its high ionic conductivity has led to intense effort to develop high energy batteries. The two most highly conductive members of the Na_2_O-Al_2_O_3_ system are Na_2_O·1Al_2_O_3_ (β-alumina) and Na_2_O·5Al_2_O_3_ (β'-alumina). The electrical conductivity of polycrystalline β'-alumina is approximately 0.2 S cm^–1^ at 300 °C [128]. NAS batteries are expected to have high energy density, high charge/discharge efficiency, and long cycle life. Therefore, early studies on this battery were focused on exploiting these properties for application in electric vehicles in Europe and the United States. However, batteries with the high energy density required for electric vehicles have not yet been developed with this system. NAS batteries have also been considered as promising candidates for stationary storage applications. Significant achievements have been made in such applications, especially in Japan [129,130]. Figure 36 shows a cross-sectional view of a NAS battery in which molten sodium metal serves as the negative electrode and is contained inside a β'-alumina tube with a porous metal wick. The β' alumina tube is sealed with glass solder to an α-alumina header that electrically insulates it from the negative electrode. A pre-cast matrix of graphite felt impregnated with molten sulfur, which serves as the positive electrode, is placed in the gap between the β’-alumina tube and the cell container. NGK Insulators, Ltd. had commercialized NAS batteries in Japan in 2002. Their standard 2 MW and 12 MWh (forty 50 kW units) system has a guaranteed service life of 15 years (2500 cycles at 100% discharge depth, 4500 cycles at 90% discharge depth). The NAS based batteries installed worldwide reached 530 MW in 2012, according to NGK Insulators.

## 10. Conclusions and future developments

In this article, the author reviewed the development of solid state ionics from its basic science to possible applications, from the viewpoint of discovery of new materials. If we look back through the last 70-year history of solid state ionics research, Japan was a major contributing country that played an active role in the discovery, theoretical modeling, and applications of new materials. The development of solid state ionics can be illustrated by comparing the popular topics today with those in the early days: 24 papers were presented at the first Solid State Ionics Symposium in Japan, organized in Nagoya in 1972. The main research topics in the symposium were the properties of the typical highly ion conducting solid electrolytes and ionic and electron mixed conductors, most of which were already known, with an addition of newly discovered fast ion conductors, then, such as β-alumina and RbAg_4_I_5_. In contrast, the 38th Solid State Ionics Symposium in Japan in 2012 mostly concentrated on materials for lithium batteries and fuel cells, where research into new electrode materials and electrolytes is a key for the development of high energy and high power density secondary batteries for electric vehicle applications. In particular, electrode materials with high ion diffusivity should be developed to obtain high specific power density. SOFCs are one of the high-efficiency energy-conversion systems consisting of electrolytes and electrode materials developed in the field of solid state ionics. Thus, fundamental studies on the structure and properties of solid state ionic materials are essential to discover new materials for such batteries and energy-conversion devices. In addition, although ion migration in nanoelectronic devices, such as CMOS devices, has been considered a detrimental issue best avoided, utilization of local ion migration and resultant local redox reaction is growing and expanding very rapidly, receiving much attention as one of most advanced topics to realize nanoelectronic devices controlled by ionic motion.

Research activity in solid state ionics has been dependent on the discovery of new solid ionic materials; novel materials open the door of a new material science. For half a century, many new solid state ionics materials have come from Japan, as reviewed here, and the author hopes for more to come in future. Finally, solid state ionics originated in Europe, and an excellent review of the development of solid state ionics there, from foundation up to the present day, was presented by Funke [131].

## Disclosure statement

No potential conflict of interest was reported by the author.
